# Palaeoecological data indicates land-use changes across Europe linked to spatial heterogeneity in mortality during the Black Death pandemic

**DOI:** 10.1038/s41559-021-01652-4

**Published:** 2022-02-10

**Authors:** A. Izdebski, P. Guzowski, R. Poniat, L. Masci, J. Palli, C. Vignola, M. Bauch, C. Cocozza, R. Fernandes, F. C. Ljungqvist, T. Newfield, A. Seim, D. Abel-Schaad, F. Alba-Sánchez, L. Björkman, A. Brauer, A. Brown, S. Czerwiński, A. Ejarque, M. Fiłoc, A. Florenzano, E. D. Fredh, R. Fyfe, N. Jasiunas, P. Kołaczek, K. Kouli, R. Kozáková, M. Kupryjanowicz, P. Lagerås, M. Lamentowicz, M. Lindbladh, J. A. López-Sáez, R. Luelmo-Lautenschlaeger, K. Marcisz, F. Mazier, S. Mensing, A. M. Mercuri, K. Milecka, Y. Miras, A. M. Noryśkiewicz, E. Novenko, M. Obremska, S. Panajiotidis, M. L. Papadopoulou, A. Pędziszewska, S. Pérez-Díaz, G. Piovesan, A. Pluskowski, P. Pokorny, A. Poska, T. Reitalu, M. Rösch, L. Sadori, C. Sá Ferreira, D. Sebag, M. Słowiński, M. Stančikaitė, N. Stivrins, I. Tunno, S. Veski, A. Wacnik, A. Masi

**Affiliations:** 1 Max Planck Institute for the Science of Human History, Jena, Germany; 2 Institute of History, Jagiellonian University in Krakow, Krakow, Poland; 3 Faculty of History and International Relations, University of Bialystok, Bialystok, Poland; 4 Department of Earth Science, Sapienza University of Rome, Rome, Italy; 5 Department of Environmental Biology, Sapienza University of Rome, Rome, Italy; 6 Department of Agriculture and Forest Sciences (Dafne), University of Tuscia, Viterbo, Italy; 7 Department of Ecological and Biological Sciences (Deb), University of Tuscia, Viterbo, Italy; 8 Leibniz Institute for the History and Culture of Eastern Europe (GWZO), Leipzig, Germany; 9 ArchaeoBioCenter, Ludwig-Maximilians-Universität München, München, Germany; 10 School of Archaeology, University of Oxford, Oxford, UK; 11 Faculty of Arts, Masaryk University, Brno, Czech Republic; 12 Department of History, Stockholm University, Stockholm, Sweden; 13 Bolin Centre for Climate Research, Stockholm University, Stockholm, Sweden; 14 Swedish Collegium for Advanced Study, Uppsala, Sweden; 15 Department of History, Georgetown University, Washington DC, USA; 16 Department of Biology, Georgetown University, Washington DC, USA; 17 Chair of Forest Growth and Dendroecology, Institute of Forest Sciences, Albert-Ludwigs-University Freiburg, Freiburg, Germany; 18 Department of Botany, University of Innsbruck, Innsbruck, Austria; 19 Department of Botany, University of Granada, Granada, Spain; 20 Viscum Pollenanalys & Miljöhistoria, Nässjö, Sweden; 21 GFZ-German Research Centre for Geosciences, Section Climate Dynamics and Landscape Evolution, Potsdam, Germany; 22 Institute of Geosciences, University of Potsdam, Potsdam, Germany; 23 Wessex Archaeology, Portway House, Salisbury, UK; 24 Department of Archaeology, University of Reading, Reading, UK; 25 Climate Change Ecology Research Unit, Adam Mickiewicz University, Poznań, Poland; 26 CNRS, Université Clermont Auvergne, GEOLAB, Clermont-Ferrand, France; 27 ISEM, UMR 5554, Université Montpellier, CNRS, EPHE, IRD, Montpellier, France; 28 Department of Palaeobiology, Faculty of Biology, University of Białystok, Białystok, Poland; 29 Laboratory of Palynology and Palaeobotany, Department of Life Sciences, University of Modena and Reggio Emilia, Modena, Italy; 30 Museum of Archaeology, University of Stavanger, Stavanger, Norway; 31 School of Geography, Earth and Environmental Science, University of Plymouth, Plymouth, UK; 32 Department of Geography, University of Latvia, Riga, Latvia; 33 Department of Geology and Geoenvironment, National and Kapodistrian University of Athens, Athens, Greece; 34 Institute of Archeology, Academy of Sciences of the Czech Republic, Prague, Czech Republic; 35 The Archaeologists, National Historical Museums, Lund, Sweden; 36 Southern Swedish Forest Research Centre, Swedish University of Agricultural Sciences, Alnarp, Sweden; 37 Environmental Archaeology Research Group, Institute of History, CSIC, Madrid, Spain; 38 Department of Geography, Universidad Autónoma de Madrid, Madrid, Spain; 39 Department of Environmental Geography, GEODE UMR 5602, Jean Jaurès University, Toulouse, France; 40 Department of Geography, University of Nevada, Reno, USA; 41 Anthropocene Research Unit, Faculty of Geographical and Geological Sciences, Adam Mickiewicz University, Poznań, Poland; 42 CNRS, HNHP UMR 7194, Muséum National d’Histoire Naturelle, Institut de Paléontologie Humaine, Paris, France; 43 Institute of Archaeology, Faculty of History, Nicolaus Copernicus University, Toruń, Poland; 44 Centre for Climate Change Research, Nicolaus Copernicus University, Toruń, Poland; 45 Faculty of Geography, Lomonosov Moscow State University, Moscow, Russia; 46 Department of Quaternary Research, Institute of Geography Russian Academy of Science, Moscow, Russia; 47 Institute of Geological Sciences, Polish Academy of Sciences, Warsaw, Poland; 48 Laboratory of Forest Botany-Geobotany, School of Forestry and Natural Environment, Aristotle University of Thessaloniki, Thessaloniki, Greece; 49 Institute of Geography, University of Cologne, Cologne, Germany; 50 Laboratory of Palaeoecology and Archaeobotany, Department of Plant Ecology, Faculty of Biology, University of Gdańsk, Gdańsk, Poland; 51 Department of Geography, Urban and Regional Planning, Universidad de Cantabria, Santander, Spain; 52 Centre for Theoretical Study, Charles University and Academy of Sciences of the Czech Republic, Prague, Czech Republic; 53 Department of Geology, Tallinn University of Technology, Tallinn, Estonia; 54 Department of Physical Geography and Ecosystem Science, Lund University, Lund, Sweden; 55 Institute of Ecology and Earth Sciences, University of Tartu, Tartu, Estonia; 56 Department of Pre- and Early History and West Asian Archaeology, University of Heidelberg, Heidelberg, Germany; 57 School of Natural and Built Environment, Queen’s University, Belfast, Northern Ireland; 58 IFP Energies Nouvelles, Earth Sciences and Environmental Technologies Division, Rueil-Malmaison, Rueil-Malmaison, France; 59 Past Landscape Dynamics Laboratory, Institute of Geography and Spatial Organization, Polish Academy of Sciences, Warsaw, Poland; 60 Nature Research Centre, Institute of Geology and Geography, Vilnius, Lithuania; 61 Institute of Latvian History, University of Latvia, Riga, Latvia; 62 Center for Accelerator Mass Spectrometry (CAMS), Lawrence Livermore National Laboratory, Lawrence, CA USA; 63 W. Szafer Institute of Botany, Polish Academy of Sciences, Kraków, Poland

**Keywords:** Environmental economics, Environmental studies, History, Palaeoclimate, Socioeconomic scenarios

## Abstract

The Black Death (1347–1352 ce) is the most renowned pandemic in human history, believed by many to have killed half of Europe’s population. However, despite advances in ancient DNA research that conclusively identified the pandemic’s causative agent (bacterium *Yersinia pestis*), our knowledge of the Black Death remains limited, based primarily on qualitative remarks in medieval written sources available for some areas of Western Europe. Here, we remedy this situation by applying a pioneering new approach, ‘big data palaeoecology’, which, starting from palynological data, evaluates the scale of the Black Death’s mortality on a regional scale across Europe. We collected pollen data on landscape change from 261 radiocarbon-dated coring sites (lakes and wetlands) located across 19 modern-day European countries. We used two independent methods of analysis to evaluate whether the changes we see in the landscape at the time of the Black Death agree with the hypothesis that a large portion of the population, upwards of half, died within a few years in the 21 historical regions we studied. While we can confirm that the Black Death had a devastating impact in some regions, we found that it had negligible or no impact in others. These inter-regional differences in the Black Death’s mortality across Europe demonstrate the significance of cultural, ecological, economic, societal and climatic factors that mediated the dissemination and impact of the disease. The complex interplay of these factors, along with the historical ecology of plague, should be a focus of future research on historical pandemics.

## Abstract (web summary)

Historical accounts of the mortality outcomes of the Black Death plague pandemic are variable across Europe, with much higher death tolls suggested in some areas than others. Here the authors use a ‘big data palaeoecology’ approach to show that land use change following the pandemic was spatially variable across Europe, confirming heterogeneous responses with empirical data.

## Main

Few doubt that the mid-fourteenth-century Afro-Eurasian plague pandemic, the Black Death, killed tens of millions of people. In western Asia and Europe, where its spread and mortality are best understood, upwards of 50% of the population is thought to have died within approximately 5 years^1–4^. Whole-genome sequencing confirms the pandemic as a novel introduction of the zoonotic bacterium *Yersinia pestis*^5,6^. Yet, despite advances in palaeogenetics and generations of written-source-based research on the cultural and economic transformations plague is credited with accelerating, from the Renaissance to the ‘Great Divergence’^7,8^, much about the Black Death’s spread and demographic impact remains poorly understood. The regionality of the plague’s mortality is particularly underexplored, owing to the availability of written sources and the limits of traditional historical methods. Here we pioneer a new approach, big data palaeoecology (BDP), that leverages the field of palynology to evaluate the demographic impact of the Black Death on a regional scale across Europe, independent of written sources and traditional archaeological material. Our analysis of 1,634 pollen samples from 261 sites, reflecting landscape change and agricultural activities, demonstrates Black Death mortality was far more spatially heterogeneous than previously recognized. Strikingly, BDP provides independent confirmation of the devastating toll of the Black Death reflected in written sources in some European regions, while establishing conclusively that the Black Death did not affect all regions equally. We attribute this mortality variation to cultural, ecological, economic, societal and climatic factors, which influenced *Y. pestis* dissemination and prevalence, generating regionally unique outcomes.

## Estimating Black Death mortality

Multidisciplinary studies are redefining the Black Death. In recent years, palaeogeneticists have confirmed the pandemic’s *Y. pestis* identity and established that the outbreak seeded novel plague reservoirs in Europe^5,6^. Archaeologists and historians meanwhile have begun to put sub-Saharan Africa on the Black Death map^9^, to fill in lacunae in our understanding of the pandemic’s Mediterranean and European spread^10,11^, and to explore the pandemic’s origins in central Asia and dissemination in east Asia, drawing on evolutionary biology and palaeogenetics^12^. But while multiple disciplines have reassessed the pandemic’s spatiotemporality, its mortality—estimates of which have drawn attention to the Black Death for centuries—remains underexplored and limited almost entirely to unidisciplinary, written-source-based approaches.

Medieval mortality data are scarce and highly fragmentary. Nineteenth-century historians of the Black Death based their assertion that the pandemic claimed 25% or more of European lives on assessments of qualitative narrative sources^13^. Since the mid-twentieth century, historians have painstakingly built-up multiple instructive case studies of Black Death mortality for regions comparatively well-endowed with administrative sources allowing for statistical analysis (for example, regions of England, France, Italy and the Netherlands)^14–16^. Some of these case studies have argued for a death toll in the range of 50% or more. These numbers have been increasingly considered representative of the Black Death’s broader mortality^1–4,9,12,14^. Although it has been suggested that regional variation characterized the demographic crisis the Black Death caused, little evidence—historical, archaeological or environmental—has been employed to substantiate such thinking^8,17,18^. As a result, case studies from better documented regions have been employed as proxies for Black Death mortality in European regions where direct evidence for the pandemic is nonexistent (for example Bohemia and Finland), slight (single sentences or vague passages; for example Moravia, Hungary and Scotland) or available but neither detailed nor quantitative (most regions, including areas of England, France, Italy and the Netherlands)^1^.

By treating case studies of relatively well-documented regions as predictive of the pandemic’s death toll in all regions the pandemic touched, histories of the Black Death implicitly rest on the untested assumption that plague mortality was uniform across regions regardless of local cultural, ecological, economic and societal contexts, and therefore that the prevalence of *Y. pestis*, an ecologically and epidemiologically complex disease^19^, was comparable across Europe. This methodology has lent itself to estimates of an aggregate European death toll upwards of around 50% (approximately 50 million deaths)^1,4^, though studies accounting for regional source scarcities have estimated mortality to have fallen below that mark^8,17,20^. Problematically, many of the quantitative sources drawn upon to build cases studies of Black Death mortality relate to urban contexts, which owing to their crowding, generally poor sanitation and quite possibly heavier disease burdens, may have suffered higher plague mortality than rural areas^21,22^, and in the mid-fourteenth century upwards of 75–95% of the population of every European region was rural^23^. Although every pandemic, plague or not, is distinct, a Black Death mortality across Europe of approximately 50% vastly exceeds the demographic losses sustained during the third plague pandemic in the late nineteenth century—the plague pandemic for which the most mortality data exists—including in China and India, which were then severely affected^24,25^.

## BDP and Black Death mortality

**Fig. 1:**
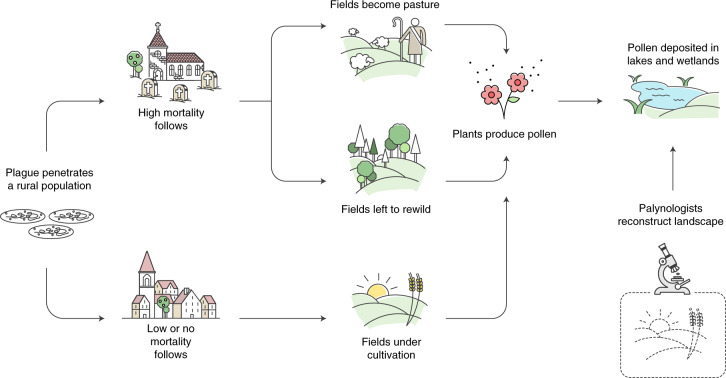
The BDP approach to verifying Black Death mortality levels. Credit: A.I., T.N., Hans Sell and Michelle O’Reilly.

**Fig. 2:**
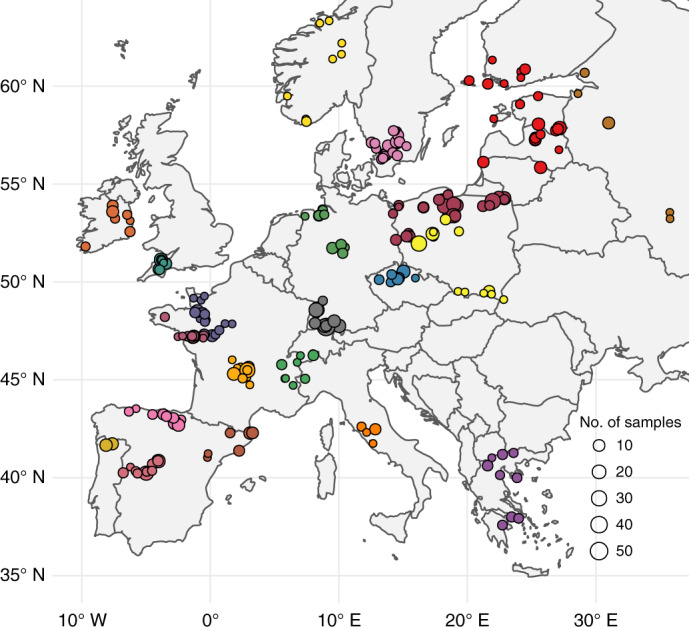
Location of pollen-analysed sediment cores used in this study. Circle size reflects the number of samples per site for the period of 1250–1450 ce, different colours reflect division of sites into regional clusters for the purpose of the analysis presented in Fig. 3.

Here, we pioneer an alternative approach (discussed in detail in Methods). BDP allows us to evaluate the Black Death’s mortality across Europe using quantitative palaeoenvironmental datasets, which can be employed for spatial statistical comparisons (Fig. 1). Our dataset consists of fossil pollen counts from 261 radiocarbon-dated sediment cores from 19 present-day European countries (Fig. 2; Supplementary Data 1). Pollen data can be used to assess past demographic dynamics as human pressure on the landscape in the pre-industrial period was directly dependent on the availability of rural labour. We focus on the period between 1250 and 1450 ce (comparing 100 years before and after the Black Death; 100 years representing roughly four generations, a time period during which pre-industrial populations could not recover from potentially high plague mortality^23^) for which 1,634 pollen-analysed sediment samples are available (Supplementary Data 2).

**Extended Data Fig. 1:**
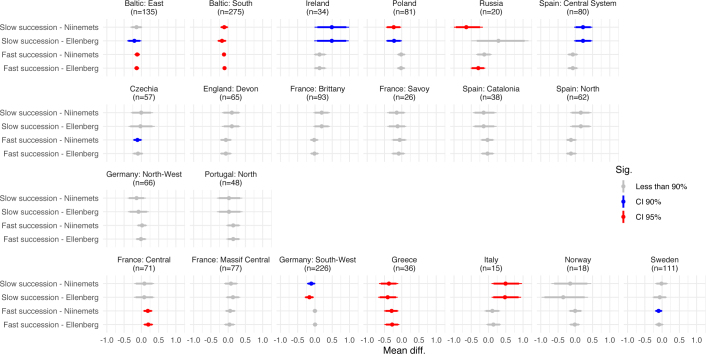
**Region-by-region comparison of Ellenberg and Niinemets indicators**.

**Extended Data Fig. 2:**
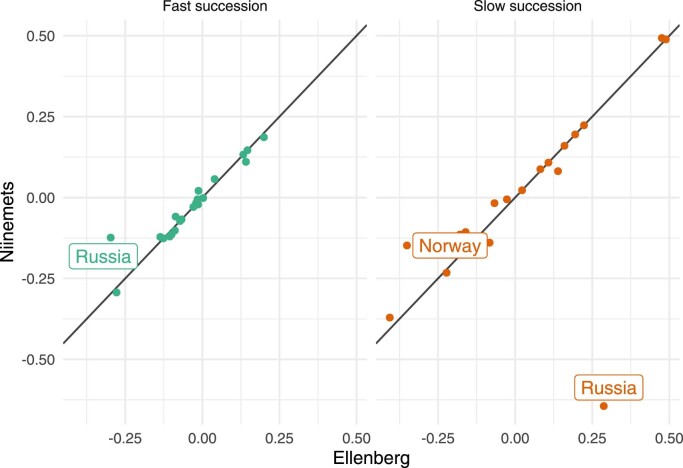
**Outlier analysis of Ellenberg and Niinemets indicators**.

In order to compare trends between different European bioclimatic zones, we assembled pollen types (taxa) representing different plants from species to family level into four standardized summary indicators, based on their subsistence value for human economy or their ecological needs as reflected in two major ecological indices, the Ellenberg light indicator and Niinemets and Valladares shade tolerance scale: (1) cereals; (2) herding (pasturelands); (3) fast forest secondary succession (pioneer shrubs and trees growing on former fields/pastures within 5–10 years of abandonment); (4) slow forest secondary succession (mature–late successional woodland on abandoned fields/pastures). We discuss the results of the Ellenberg-based indicators below, but the Niinemets-based indicators yielded the same results in 19 out of 21 regions and the differences do not bear on our discussion (Extended Data Figs. 1 and 2).

## Results

### Validity test using Sweden and Poland

**Fig. 3:**
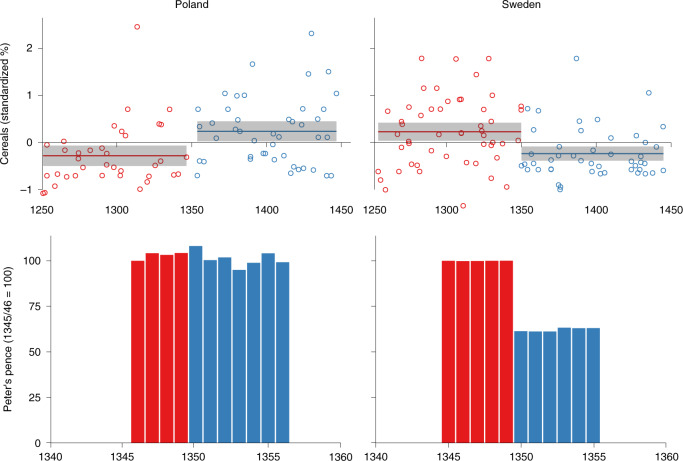
Comparison of historical and palaeoenvironmental indicators of the demographic impact of the Black Death in Poland and Sweden. Top panel, pollen data (cereal pollen, standardized percentage values from individual sites and 100-yr mean with standard deviation), see Supplementary Data 2. Bottom panel, Peter’s pence tax^27–29^.

We validated our BDP approach by examining two well-studied, but contrasting, regional case studies of the Black Death’s mortality, in Sweden and Poland. An earlier multidisciplinary analysis discovered significant contraction in cereal cultivation, as well as a more general economic and demographic decline, in the uplands of southern Sweden following the Black Death^26^. By contrast, historians have long demonstrated that central Europe, particularly Poland, experienced economic growth over the fourteenth century, related to the centralization of royal power following a period of partition, few wars in the central provinces of the country, large-scale colonization of uncultivated lands and the development of cities^27^. Our BDP approach independently corroborates these trajectories. We validate the ability of our pollen datasets to reflect the extent of the Black Death’s mortality by comparing them to historical data of national tax payments made to the pope (Peter’s pence)^28–30^ (Fig. 3). This validity test lends further support to our primary focus in this study on cereal cultivation, as argued in the Methods.

### Four major scenarios of landscape and demographic change

**Fig. 4:**
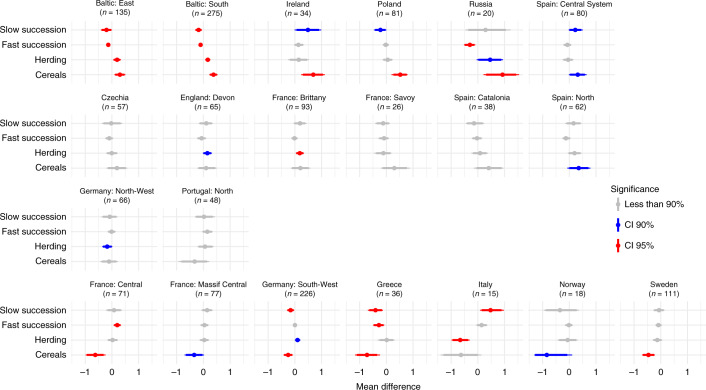
Regional-scale changes following Black Death in four BDP pollen indicators. Difference between the means of 100-yr periods of 1250–1350 and 1351–1450 ce, with the standard deviation. Statistical significance is based on bootstrap estimates. The indicators are presented in four rows from statistically significant increases in standardized mean percentages of cereal pollen (top) to statistically significant declines in standardized mean percentages of cereal pollen (bottom).

**Extended Data Fig. 3:**
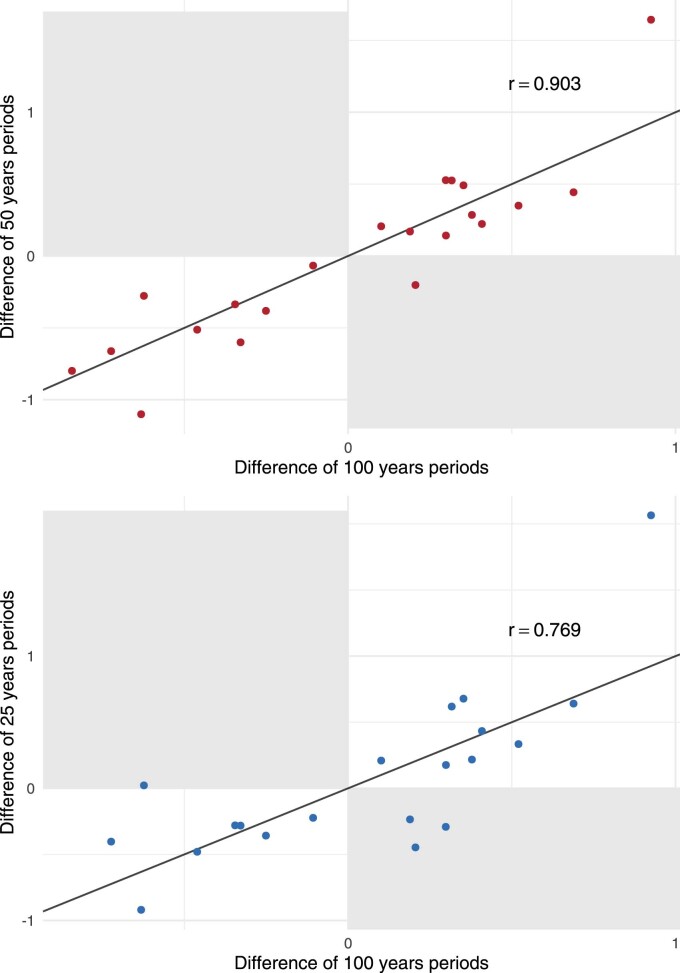
Differences and similarities in the changes in the cereal pollen indicator between 100-yr periods of analysis versus 50-yr and 25-yr periods respectively. Points displayed in white areas represent regions where the direction of changes was the same over the longer versus shorter periods of time. Points displayed in grey areas represent regions where the direction of changes was different over the longer versus shorter periods of time. Regions showing such differences have been named. Based on Supplementary Figs. 1 and 2.

**Extended Data Fig. 4:**
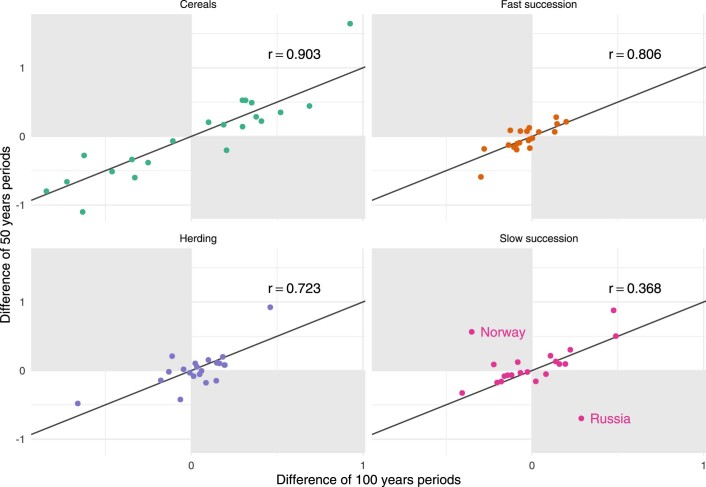
Differences and similarities in the changes in the BDP pollen indicators between 100-yr and 50-yr periods of analysis. Points displayed in white areas represent regions where the direction of changes was the same over the longer versus shorter periods of time. Points displayed in grey areas represent regions where the direction of changes was different over the longer versus shorter periods of time. Based on Supplementary Fig. 1. Please note: Norway and Russia were the only regions that showed significant differences between Ellenberg and Niinemets indicators, most strongly for the slow succession indicator. Based on Supplementary Fig. 1.

**Extended Data Fig. 5:**
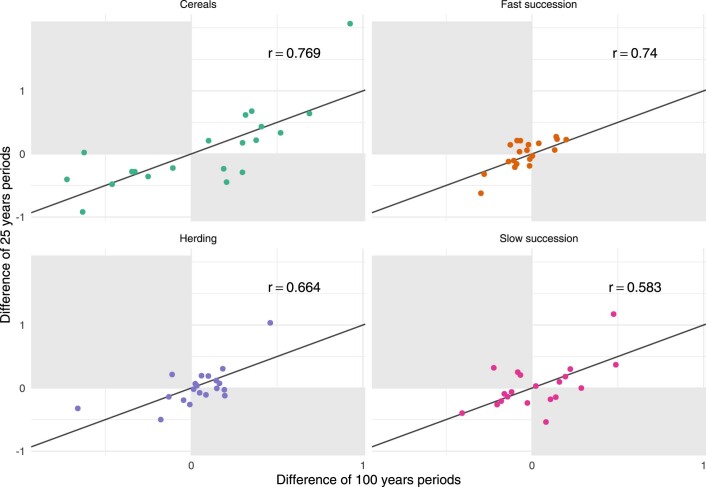
Differences and similarities in the changes in the BDP pollen indicators between 100-yr and 25-yr periods of analysis. Points displayed in white areas represent regions where the direction of changes was the same over the longer versus shorter periods of time. Points displayed in grey areas represent regions where the direction of changes was different over the longer versus shorter periods of time. Based on Supplementary Fig. 2.

Having validated our approach, we analysed the pollen dataset for all of Europe. We focused on contrasting the four summary pollen indicator values on a regional scale for subperiods of 100 years before and after the Black Death. While we discuss the results of this analysis here (Fig. 4), our complimentary data presents 50-year (1301–1350 to 1351–1400) and 25-year (1325–1350 to 1351–1375) period analyses (Supplementary Figs. 1 and 2). For cereals, they returned the same results in terms of direction of change for 20 out of 21 regions for 50-year and 16 out of 19 regions for 25-year analyses, confirming the robustness of our conclusions (Extended Data Fig. 3); the results for other indicators are also highly similar between different periods of analysis (Extended Data Figs. 4 and 5).

BDP employs bootstrapping to evaluate statistical significance of the differences between the pre- and post-Black Death subperiods on a regional level. In this way, we identified four scenarios of post-Black Death agricultural change based on the cereal pollen indicator (Fig. 4): (1) substantial and statistically significant increase, reflecting arable expansion and limited Black Death mortality (top panel); (2) modest and statistically insignificant increase, suggesting stability or slow-paced agrarian growth (upper middle panel); (3) modest and statistically insignificant decrease, suggesting stagnation or some contraction of agrarian activities, possibly stemming from more limited demographic losses or economic disruption caused by the plague (lower middle panel); (4) substantial and statistically significant decrease, reflecting arable contraction and pronounced Black Death mortality (bottom panel). Scenarios 1 and 2 falsify the theory that Black Death mortality was significant everywhere. Scenario 3 and 4 demonstrate Black Death mortality was devastating in some regions.

Changes in the herding indicator reveal trajectories similar to those of cereals in most cases or no change. We discovered only one region (southwest Germany) where there occurred a statistically significant decline in cultivation in parallel with a statistically significant increase in herding (some indication of this is also apparent in Greece), suggesting a shift to livestock production related to agrarian labour shortages and less demand for grain^31^. The fast forest succession indicator increases in a statistically significant way in central Italy and central France, confirming field abandonment. In central Italy, this is accompanied by discernible reforestation (statistically significant increase in the slow forest succession indicator), attesting to significant regional Black Death mortality and slow demographic and economic recovery.

**Fig. 5:**
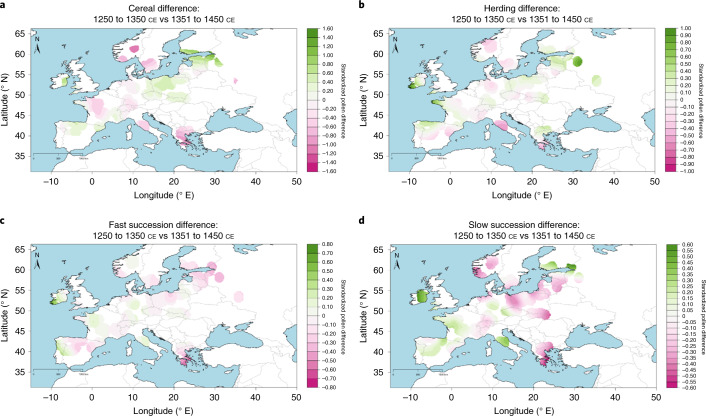
Spatial extrapolation showing 1250–1350 ce versus 1351–1450 ce temporal variation in the BDP pollen indicators. **a**–**d**, Cereal (**a**), herding (**b**), fast succession (**c**) and slow succession (**d**). Based on results from Supplementary Fig. 3.

To confirm the robustness of our results, BDP combines statistical with independent spatial approaches (Fig. 5). The latter yielded results identical for all four BDP indicators to our statistical approach in our 100-year period analysis as well as in our 50-year (1301–1350 ce to 1351–1400 ce) and 25-year (1325–1350 ce to 1351–1375 ce) period analyses (Supplementary Figs. 4–7).

## Discussion

### The Black Death was a diverse and entangled phenomenon

**Fig. 6:**
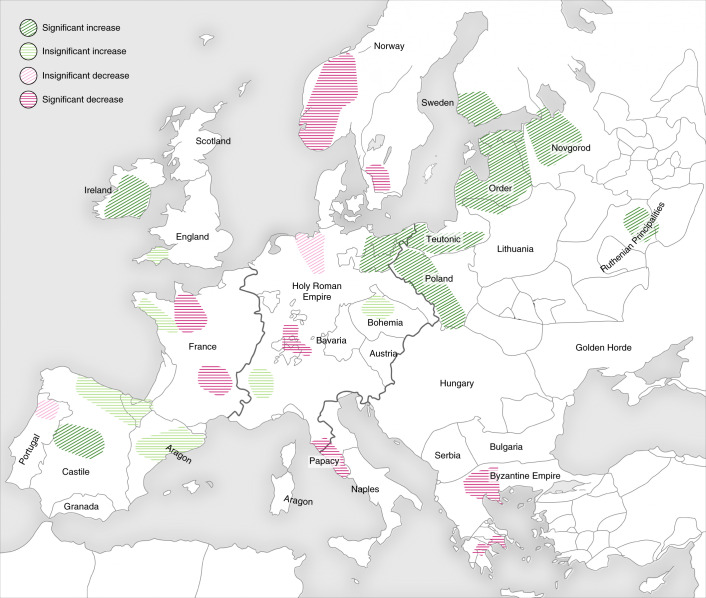
BDP-determined regional scenarios of Black Death demographic impact. Colours reflect centennial-scale changes in the cereal pollen indicators from Fig. 2. Background map with political borders of fourteenth-century Europe. Credit: Hans Sell, Michelle O’Reilly and A.I.

Figure 6 visualizes the spatial distribution of the four trajectories of post-Black Death landscape change from Fig. 4, demonstrating that the Black Death’s mortality varied significantly between European regions. The pandemic was immensely destructive in some areas, but in others it had a far lighter touch. Strikingly, BDP identifies a sharp agricultural decline in several regions of Europe, independently corroborating analyses of historical sources that suggest high mortality in regions of Scandinavia, France, western Germany, Greece and central Italy^1^, and lending further validation to our approach. At the same time, there is much evidence for continuity and uninterrupted agricultural growth in central and eastern Europe, and several regions of western Europe, particularly in Ireland and Iberia. In this way, BDP invalidates histories of the Black Death that assume *Y. pestis* was uniformly prevalent, or nearly so, across Europe and that the pandemic had a devastating demographic impact everywhere.

While we have centred our study on estimating the impact of the Black Death on the landscape, plague recurred in several regions within the 100-, 50- and 25-year periods of our analyses. It is not implausible that some recurrences, notably the so-called *pestis secunda* of the late 1350s and early 1360s^32^, could have been more devastating in a few regions we studied than the Black Death and that some of the arable contraction we detect may be attributable to both the Black Death and early second-pandemic recurrences. That the results of our 100-, 50- and 25-year period analyses were largely uniform, strongly suggests that if early plague recurrences contributed considerably to the landscape change, the earliest of those recurrences, occurring within the first 25 years, were the most significant. Of course, if the *pestis secunda* partially accounts for the arable contraction and forest succession shown here for parts of Europe, our results would further call into question the demographic toll of the Black Death, even where we have discerned significant arable contraction, as in parts of France, Italy and Scandinavia. At the same time, if we are to include the earliest recurrences, where we have discerned little change or arable expansion, the significance of both the Black Death and those earliest recurrences is limited. These remarks aside, our approach has shown conclusively that the Black Death did not significantly alter land use everywhere or affect all regions equally. The significant variability in mortality that our BDP approach identifies remains to be explained, but local cultural, demographic, economic, environmental and societal contexts would have influenced *Y. pestis* prevalence, morbidity and mortality. Ongoing transformations of rural economy in many European regions would have also modified the plague mortality, or—to an extent—the amplified impact it had on the landscape. Importantly, regional population densities cannot explain the complex landscape dynamics we discern (as visualized in Supplementary Fig. 8), nor the geographical location of the 261 coring sites (see Supplementary Fig. 9, showing the averaged elevation of our sites). The spread of the pandemic depended on numerous factors, which would have generated compound effects, feedback loops and regionally unique outcomes. Plague is an ecologically and epidemiologically complex zoonotic disease, maintained by sylvatic rodents and their fleas, and transmittable to and between people via multiple pathways, including commensal and sylvatic rodent flea bites, respiratory secretions, direct contact with infected animals, human ectoparasites (fleas and lice) and fomites^19^. The behaviour of *Y. pestis* hosts and vectors, and their capacity to efficiently transmit the pathogen, is partly constrained by complex interactions with seasonal climate variability and local ecological conditions, both in anthropogenic and rural environments (cities, villages and fields versus mountains, forests or wetlands^32^). Regional variation in population density and distribution, ectoparasite burdens, living conditions, and commensal rodent populations and their fleas, undoubtedly mattered.

Local climatic contexts were also shown to have strongly determined third plague pandemic dynamics in Asia^33,34^. In Europe, where the Black Death spread over several years (1347–1352 for the regions considered here), different seasons and annually variable climatic conditions may have influenced *Y. pestis* prevalence and the pandemic’s mortality. Furthermore, while hypothesized links between early-fourteenth-century famines and the Black Death remain to be substantiated, and while *Y. pestis* is often lethal in lieu of antibiotics^19^, it has been shown that Black Death mortality was selective and the immunological and nutritional heterogeneity of the populations the pathogen interacted with would have ensured uneven toll^35,36^.

That climatic, cultural, ecological and economic factors shaped regional Black Death outcomes is well illustrated by the initial phases of the pandemic in Europe. Cereal trade is thought to have been instrumental for the introduction of the pandemic to Mediterranean Europe, and along established conduits of commerce and communication, ecological factors, associated contingency effects and historical path dependency mattered from the outset. Before the pandemic arrived in the Crimea, the volume of cereal trade between Italy and the Black Sea was sizable, yet blocked by embargo^11,37,38^. High demand in southern Europe for Black Sea cereals from 1345 onwards was associated with a period of excessive precipitation and cooling^39,40^ negatively affecting cereal supplies in Italy and beyond, 1345–47^8,37,41,42^. Cereal imports from Black Sea coasts resumed once the situation improved in 1347, and by early 1348 Venetian merchants had filled many Italian granaries with Black Sea produce^11,37,39,43^ and introduced plague to Europe. Plague outbreaks disseminated from major cereal ports in southern and north-western Italy from January 1348^1,44^. To the contrary, plague hardly spread in north-western Italy, which was independent from overseas cereal imports^37,43,45^. Local circumstances shaped the outcome of the pandemic on regional scales from the outset.

In summary, our BDP approach shows Black Death mortality was far more spatially heterogeneous than previously thought. This significant variation in Black Death mortality may be explained by the pathogen’s entanglement with a dynamic nexus of cultural, ecological, economic, societal and climatic factors that determined its prevalence and the pandemic’s mortality in any given region. That the pandemic was immensely destructive in some regions, but not all, falsifies the practice, not uncommon in Black Death studies, of predicting one region’s experience on the basis of another’s. Regional mortality outcomes must be reconstructed using local sources, including BDP as proxies of changes in cultural landscapes. As a few well-documented case studies of the Black Death’s destructive mortality in Europe have informed estimates of the pandemic’s mortality not only in other European regions, but also in several regions of Africa and Asia, our findings have significant implications both for the wider history of the Black Death and for how historical disease outbreaks are reconstructed.

## Methods

### Pollen-inferred landscape change and pre-industrial demography

Recently, data derived from tree rings or ice cores have been employed to approximate changes in human economic activity related to past epidemics, as well as to warfare and climatic variability^46,47^. However, none of these proxies is directly related to human demography or provides a basis to estimate variation in the Black Death’s mortality on a regional scale across Europe (to date only a single archaeological study using pottery as a proxy for demographic change on the national level, focusing on just a single country—England—has appeared^48^).

In recent years, pollen data have been proven to be closely related to demographic variability. Most importantly, detailed comparisons of historical documentary data on population trends and landscape changes as revealed by pollen data have been carried out on a local scale and a close link between changes in European pollen data and changes in European local demography over the past millennium has been demonstrated on multiple occasions, that is, during the period and region of our concern here^49,50^. A strong link between long-term demographic trends as visible in regional settlement numbers and macro-changes in land cover (deforestation/afforestation) have also been confirmed for ancient Greece^51^. Additionally, a recent publication successfully employed pollen data to test the extent of the mortality associated with the sixteenth-century Spanish and Portuguese empires’ colonization of tropical regions in the Americas and Asia^52^. However, as of now there is no method to quantify past demographic trends in absolute numbers based on palaeoecological data. Consequently, we also focus in this paper on relative changes in historical societies’ populations and test the now common idea that the Black Death caused enormous mortality across Europe (with many scholars now arguing for a mortality exceeding 30% and upwards of 50% of the population within a few years) (see also Fig. 1). Using our BDP approach, we conclude this hypothesis is not maintainable. Our evidence for demography-related landscape changes (or lack thereof) negates it.

Our main indicator is cereal pollen. In pre-industrial economies, rural labour availability (hence rural population levels) and the spatial scale of cereal cultivation were directly related. An increase in the extent and intensity of cereal cultivation—as reflected in pollen data—would have required not only a predilection and demand for cereals, but also greater availability of labour and thus population growth or significant immigration. The maintenance of existing agricultural activity, in turn, would have required relatively stable population levels^53–55^. The uniform ~50% mortality postulated for the Black Death across Europe should have resulted in a large and significant decline of cereal cultivation and parallel forest regrowth across Europe, as previously demonstrated for mid-fourteenth-century Sweden^26^ and singular sites in some regions of western Europe^56^. This result agrees with the fact that Black Death mortality could be high among people at productive age, as illustrated for England^57,58^. Moreover, even in the case of England, a comparatively commercialized and adaptive rural economy in mid-fourteenth-century Europe, the loss of 50% of the population led to a significant decline in the total area under cultivation (as documented by heterogeneous written sources)^59^. In Italy, another well-developed economy at that time, the expansion of large estates following the Black Death also did not compensate for the general loss of cereal productivity^60^. This effect, high mortality driving arable contraction, must have been yet more pronounced in more subsistence-oriented and less adaptive economies, with limited surplus production, such as in regions of the Iberian Peninsula, Germany, Sweden and particularly east-central Europe. Importantly, palaeoecological evidence for arable contraction may be indicative, to some extent, of not only rural population decline but also urban population decline in the region, as there is evidence in some areas, following the pandemic, of rural-to-urban migration, of country-dwellers repopulating urban centres^10^. Possibly less common was intraregional rural migration, as marginal lands were abandoned for better quality soils, which were more likely to remain under cultivation^26,61^.

Therefore, cereal pollen remains our most potent pollen indicator related to demographic changes in pre-industrial European societies. Other pollen indicators, reflecting rewilding and reforestation (secondary ecological succession) of cereal fields abandoned as a result of significant mortality, or the transformation of cereal fields into pastures, which required less rural labour and thus also could have been a response to high plague mortality, play a secondary role in our analysis and provide further support for our conclusions.

### BDP data collection

Existing online palynological databases (the European Pollen Database (EPD)^62^ www.europeanpollendatabase.net, and the Czech Quaternary Palynological Database (PALYCZ)^63^, https://botany.natur.cuni.cz/palycz/), as well as personal contacts of the study authors and a systematic publication search were employed to identify palynological sites in Europe reaching the required chronological and resolution quality for the study of the last millennium. In order to enable statistical analysis, we included only sites clustered in well-defined historical-geographical regions, excluding isolated sites even if the quality of a site’s data was very good. Data of sufficient quality and amount from regions for which the Black Death is well-studied, notably central and northern England and the Low Countries, is not presently available; to the best of our knowledge, for each of these regions there currently is not more than a single isolated site^56^, which does not allow for the application of statistical approaches.

In total, 261 pollen records with the average temporal resolution of 58 years and ^14^C-age control (or varve chronology), have been collected. The age–depth models of the sequences have been provided by authors in original publications, by the EPD or developed through the Clam package (version 2.3.4) of R software for the purpose of this study. The analytical protocol for pollen extraction and identification is reported in the original publications. The Pollen Sum includes all the terrestrial taxa with some exceptions based on the selection done in the original publications. The full list of sequences, exclusions from the Pollen Sum, age-depth models and full references are reported in Supplementary Data 1.

The taxa list has been normalized by applying the EPD nomenclature. In this respect, the general name Cichorioideae includes Asteraceae subf. Cichorioideae of the EPD and PALYCZ nomenclatures, which primarily refers to the fenestrate pollen of the Cichorieae tribe^64^. Ericaceae groups *Arbutus unedo*, *Calluna vulgaris*, *Vaccinium* and different *Erica* pollen types, whereas deciduous *Quercus* comprehends both *Q. robur* and *Q. cerris* pollen types^65^. Rosaceae refers to both tree and herb species of the family. Finally, *Rumex* includes *R. acetosa* type, *R. acetosella*, *R. crispus* type, *Rumex/Oxyria* and *Urtica* groups *U. dioica* type and *U. pilulifera*.

### BDP summary pollen indicators

In order to connect changes visible in the pollen data to human demographic trajectories, we assembled four summary pollen indicators that describe specific landscapes related to human activity. They reflect different degrees of demographic pressure on the landscape (cereal cultivation, pastoral activities, which are less-labour intensive than cereal cultivation, abandonment and rewilding) as well as different durations of land abandonment that might have occurred post-Black Death. Our indicators account for the fact that Europe is a continent rich in natural heritage, with a wide range of landscapes and habitats and a remarkable wealth of flora and fauna, shaped by climate, geomorphology and human activity. In order to ensure uniform interpretation of the indicators, we relied on criteria that can be applied to all European landscapes regardless of their local specificity. Cereals and herding are directly related to human activities and are barely influenced by spatial differences. More complex is the succession of natural plants with their ecological behaviour and inter-species competition. For this reason, we relied on existing quantitative indicators of plant ecology.

The Ellenberg L – light availability indicator^66^ provides a measure of sunlight availability in woodlands and consequently of tree-canopy thickness, reflecting the scale of the natural regeneration of woodland vegetation after cultivation or pasture activities^61^. Nonetheless, ecological studies have suggested that geographic and climatic variability between different European regions can influence the Ellenberg indicator system^67–71^. The original indicators were primarily designed for Central Europe^58^, but several studies developed Ellenberg indicators for other regions, reflecting the specific ecology of the selected taxa (British Isles;^72^ Czech Republic;^73^ Greece;^74^ Italy;^75^ Sweden^76^). Plants with L values between 5 and 8 are listed in the fast succession indicator, the ones with L values ranging from 1 to 4 are included in the slow succession indicator. The result is the following list:

1) **Cereals:** only cultivated cereals have been included: *Avena/Triticum* type, Cerealia type, *Hordeum* type, *Secale*. 2) **Herding** includes pastoral indicators linked to the redistribution of human pressure: *Artemisia*, Cichorioideae, *Plantago lanceolata* type, *Plantago major/media type*, *Polygonum aviculare* type, *Rumex*, *Trifolium* type, *Urtica*, *Vicia* type. 3) **Fast Succession** comprises indicators of relatively recent reforestation of cultivated land after abandonment: *Alnus*, *Betula*, *Corylus*, Ericaceae, *Fraxinus ornus*, *Juniperus*, *Picea*, *Pinus*, *Populus*, deciduous *Quercus*, Rosaceae. 4) **Slow Succession** includes indicators of secondary succession established after several decades of abandonment: *Abies*, *Carpinus betulus*, *Fagus*, *Fraxinus*, *Ostrya/Carpinus orientalis*, *Quercus ilex* type.

In order to validate the indicators overcoming the regional limits of Ellenberg values, a different subdivision has been provided following the Niinemets and Valladares shade tolerance scale for woody species of the Northern Hemisphere^77^. The subdivision of taxa in the Fast and Slow succession indicators remains the same with only three changes: *Fraxinus ornus* and *Picea* move from Fast to Slow succession and *Fraxinus* from Slow to Fast succession. Extended Data Figs. 1 and 2 show that the two groupings yield the same results, which confirms the reliability of our indicators. There is only one clear exception (Russia), with one more region where smaller-scale diversion occurs for only one indicator, Slow Succession (Norway). The different indicator behaviour results from the different attribution of *Picea* in our two sets of succession indicators: at high latitude, *Picea* characterizes the final stage of the ecological succession and hence its different attribution results in different summary indicator values in Russia for the two stages of ecological succession, fast and slow.

Please note our summary indicators are not designed to reflect the entirety of the landscape and reconstruct all of its different components. Rather, they are a means of approximating changes in the landscape related to the types of human activities, and their intensity, as much as they relate to demographic changes in human populations using and inhabiting these landscapes.

### BDP analytical statistical and spatial methods

To control for local specificity, pollen percentages of every taxon from each pollen site were standardized. From the taxa percentage in a given year the arithmetic mean calculated for the observations from the period 1250–1450 was subtracted and the result divided by the standard deviation for the 1250–1450 period. Standardized taxa results were assembled for each site into four BDP summary indicators. Since each indicator has different numbers of taxa, the sum of standardized taxa values calculated for a given year and site was divided by the number of taxa in the indicator. For the purposes of replication, this standardized pollen dataset, comprising the four indicators for each sample from each site, is available as Supplementary Data 2.

This dataset has been analysed in two ways, statistically and spatially.

For the statistical approach, standardized regional indices of landscape transformation were created for each region by calculating the average value for all sites within the region, for each of the subperiods analysed in the study (1250–1350 and 1351–1450; 1301–1350 and 1351–1400; 1325–1350 and 1351–1375). Differences between means for each subperiod were measured by the use of the bootstrapping based on 10,000 resamples. The 90% and 95% confidence intervals were estimated with the bias-corrected and accelerated method (BCa)^78^. These results are visualized in Fig. 5 for the comparison of the subperiods of 1250–1350 versus 1351–1450, and in Supplementary Figs. 4 and 6 for the comparison of the subperiods of 1300–1350 versus 1351–1400 and 1325–1350 versus 1351–1375, respectively.

For the spatial approach, we employed the Bayesian model AverageR developed within the Pandora and IsoMemo initiatives (https://pandoraapp.earth/) to map the distribution of pollen indices across Europe. AverageR is a generalized additive model that has been described previously^79^. It relies on a thin-plate regression spline^80^ to predict new, unseen data using the following model:

$$Y_{i} = {g}( {{{{\mathrm{longitude}}}},{{{\mathrm{latitude}}}}} ) + {\varepsilon}_{i}$$

Where *Y*_*i*_ is the independent variable for site *i*; *g*(longitude, latitude) is the spline smoother; and *ε*_*i*_ ∼ *N*(0, *σε*) is the error term.

The spline smoother can be written as *X* × *β* where *X* is a fixed design matrix and *β* is the parameter vector. Surface smoothing is controlled by employing a Bayesian smoothing parameter estimated from the data and trades-off bias against variance to make the optimal prediction^75^. This parameter *β* is assumed to follow a normal distribution: *β* ~ *N*(0, 1 /*δ* × *λ* × *P*), where *P* is a so-called penalty matrix of the thin plate regression spline, which penalizes second derivatives^81^. The *δ* parameter is by default set to 1 but this can be adjusted to suit smoothing needs for each application. In our study *δ* was set at 0.9 to match the preferred spatial scale of analysis for our dataset (approx. 250 to 500 km).

AverageR was employed to generate smoothed surfaces for three sets of temporal bins (1250–1350 versus 1351–1450, as well as 1300–1350 versus 1351–1400 and 1325–1350 versus 1351–1375) and for the four BDP indicators (Supplementary Figs. 3, 5 and 7). For the same indicator the difference between the two temporal bins was plotted (Fig. 5; Supplementary Figs. 4 and 6).

### Reporting Summary

Further information on research design is available in the Nature Research Reporting Summary linked to this article.

## Supplementary information

Supplementary Information Supplementary Figs. 1–9.

Reporting Summary.

Peer Review Information.

Supplementary Data Supplementary Data 1 and 2.

## Data Availability

All data generated or analysed during this study are included in this published article (as the Supplementary Data).
