# Supplementary material for: Palaeoecological data indicates land-use changes across Europe linked to spatial heterogeneity in mortality during the Black Death pandemic
Source: Nat Ecol Evol. 2022 Feb 10;6(3):297–306. doi: 10.1038/s41559-021-01652-4 (PMC8913360; doi:10.1038/s41559-021-01652-4)
Supplement: Supplementary file 1 — Supplementary Figs. 1–9. [file 41559_2021_1652_MOESM1_ESM.pdf]

nature portfolio

https://doi.org/10.1038/s41559-021-01652-4

Supplementary information

Palaeoecological data indicates land-use changes across Europe linked to spatial heterogeneity in mortality during the Black Death pandemic

In the format provided by the
authors and unedited

Big Data Palaeoecology indicates land-use changes across Europe linked to spatial heterogeneity in mortality during the Black Death pandemic

Izdebski A.[1,2], Guzowski P.[3], Poniat R.[3], Masci L.[4,5], Palli J.[6,7], Vignola C.[1,5], Bauch M.[8], Cocozza C.[1,9], Fernandes R.[1,10,11], Ljungqvist F.C.[12,13,14], Newfield T.[15,16], Seim A.[17,18], Abel-Schaad D.[19], Alba-Sánchez F.[19], Björkman L.[20], Brauer A.[21,22], Brown A.[23,24], Czerwiński S.[25], Ejarque A.[26,27], Fiłoc M.[28], Florenzano A.[29], Fredh E.D.[30], Fyfe R.[31], Jasiunas N.[32], Kołaczek P.[25], Kouli K.[33,1], Kozáková R.[34], Kupryjanowicz M.[28], Lagerås P.[35], Lamentowicz M.[25], Lindbladh M.[36], López-Sáez J.A.[37], Luelmo-Lautenschlaeger R.[37,38], Marcisz K.[25], Mazier F.[39], Mensing S.[40], Mercuri A.M.[29], Milecka K.[41], Miras Y.[42], Noryśkiewicz A. M.[43,44], Novenko E.[45,46], Obremska M.[47], Panajiotidis S.[48], Papadopoulou M.L.[49], Pędziszewska A.[50], Pérez-Díaz S.[51], Piovesan G.[7], Pluskowski A.[52], Pokorny P.[53], Poska A.[54,55], Reitalu T.[54, 56], Rösch M.[57], Sadori L.[5], Sá Ferreira C.[58], Sebag D.[59], Słowiński M.[60], Stančikaitė M.[61], Stivrins N.[33,54,62], Tunno I.[63], Veski S.[54], Wacnik A.[64], Masi A.[1,5] *

Supplementary Figures

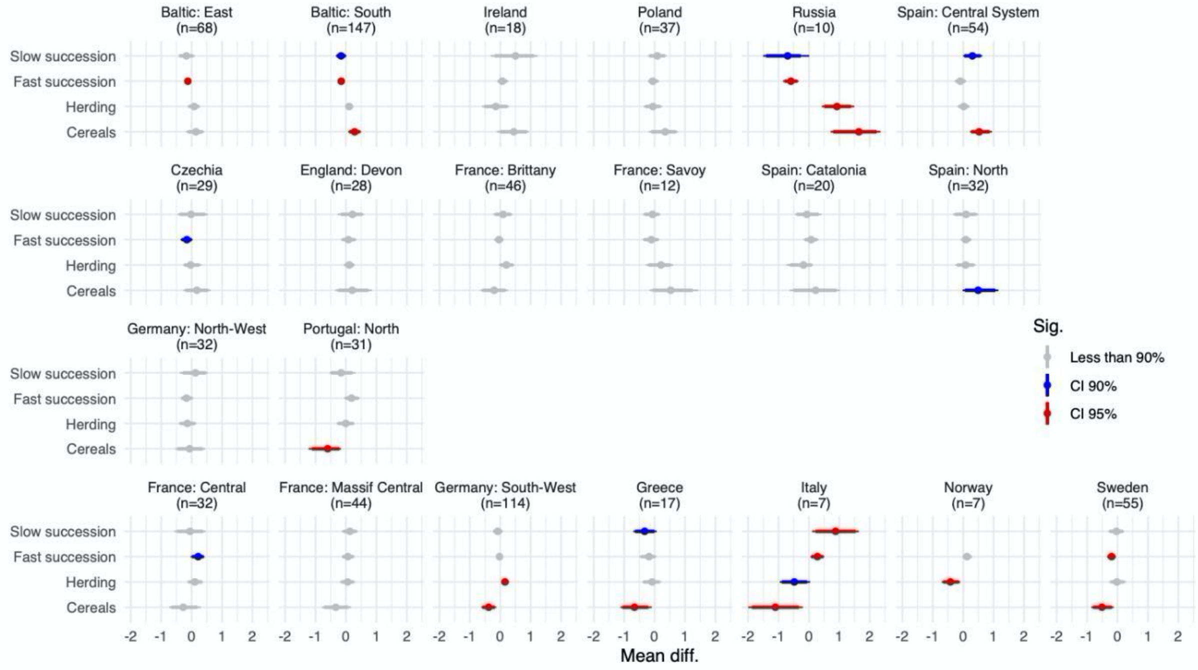

Supplementary Figure 1. ***Post-Black Death regional-scale changes in four BDP pollen indicators.*** *Difference between the means of 50-yr periods of 1301-1350 to 1351-1400 CE, with the standard deviation. Statistical significance is based on bootstrap estimates. Regions arranged as in Fig. 2.*

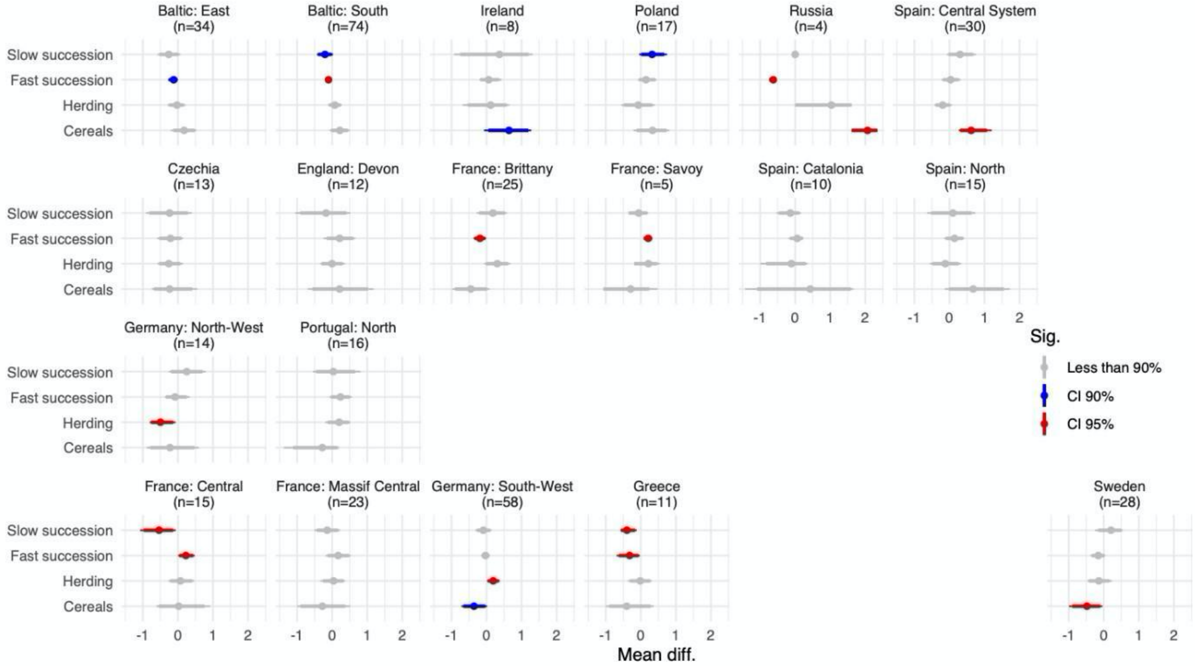

Supplementary Figure 2. ***Post-Black Death regional-scale changes in four BDP pollen indicators.*** *Difference between the means of 25-yr periods of 1325-1350 to 1351-1375 CE, with the standard deviation. Statistical significance is based on bootstrap estimates. Regions arranged as in Fig. 2.*

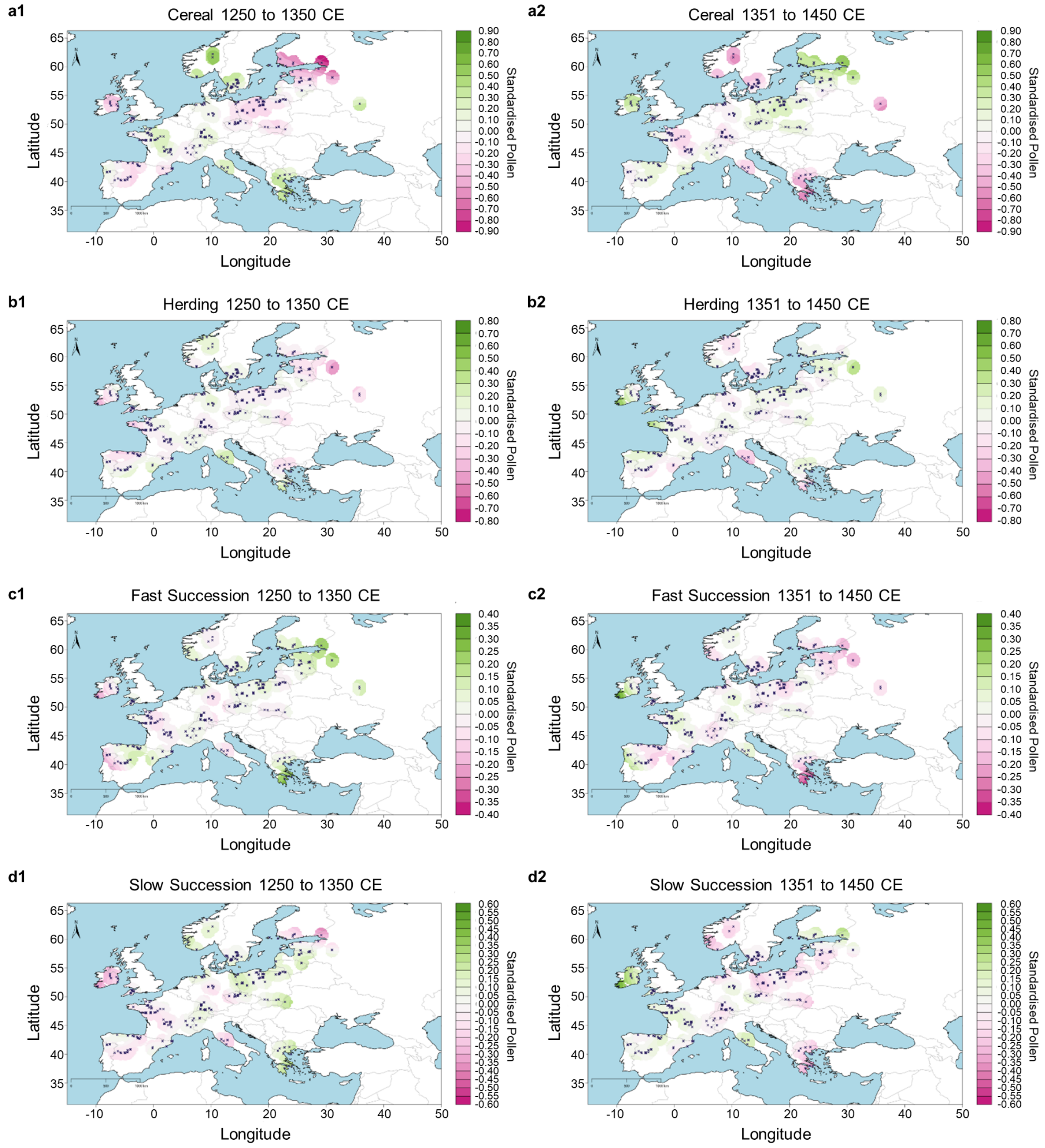

Supplementary Figure 3. Spatial extrapolation for all four indicators *c*. 100 years before and after the Black Death. **a**) Cereal 1250-1350 CE (1) and 1351-1450 CE (2) **b**) Herding 1250-1350 CE (1) and 1351-1450 CE (2) **c**) Fast Succession 1250-1350 CE (1) and 1351-1450 CE (2) **d**) Slow Succession 1250-1350 CE (1) and 1351-1450 CE (2).

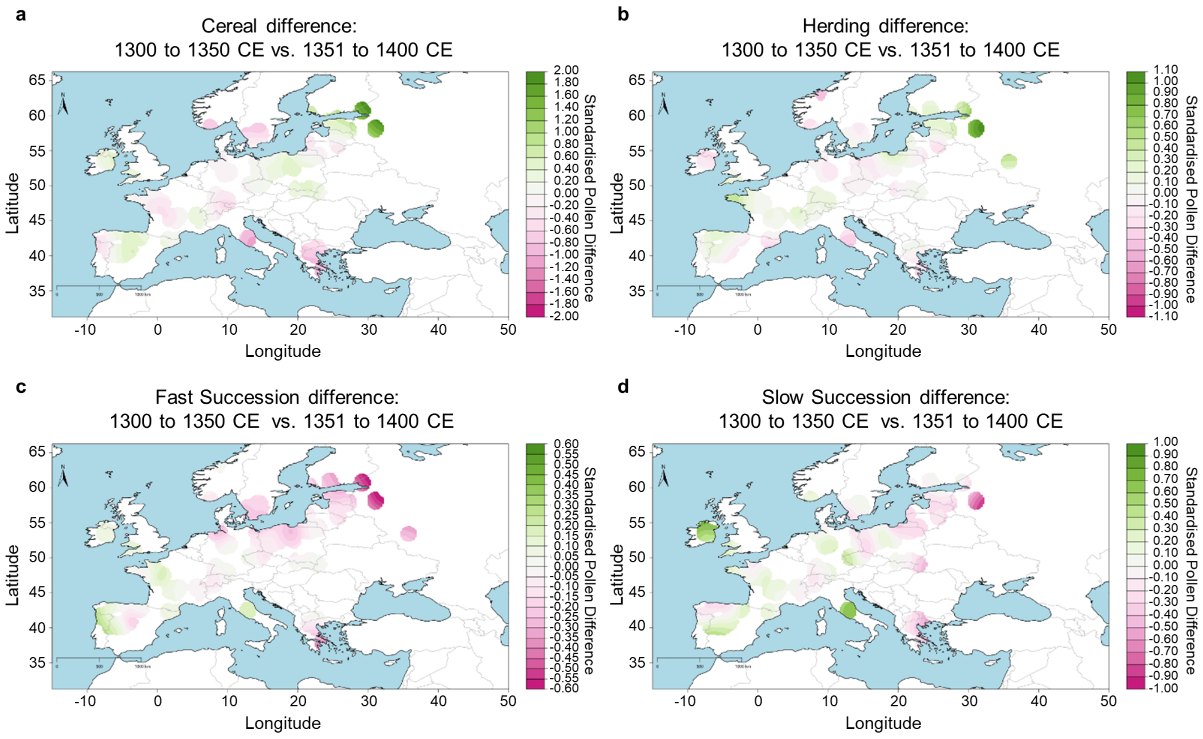

Supplementary Figure 4. ***Spatial extrapolation showing*** *1301-1350 vs 1351-1400* ***CE temporal variation in the BDP pollen indicators a****) Cereal* ***b****) Herding* ***c****) Fast Succession* ***d****) Slow Succession. Based on results from Supplementary Fig. 5.*

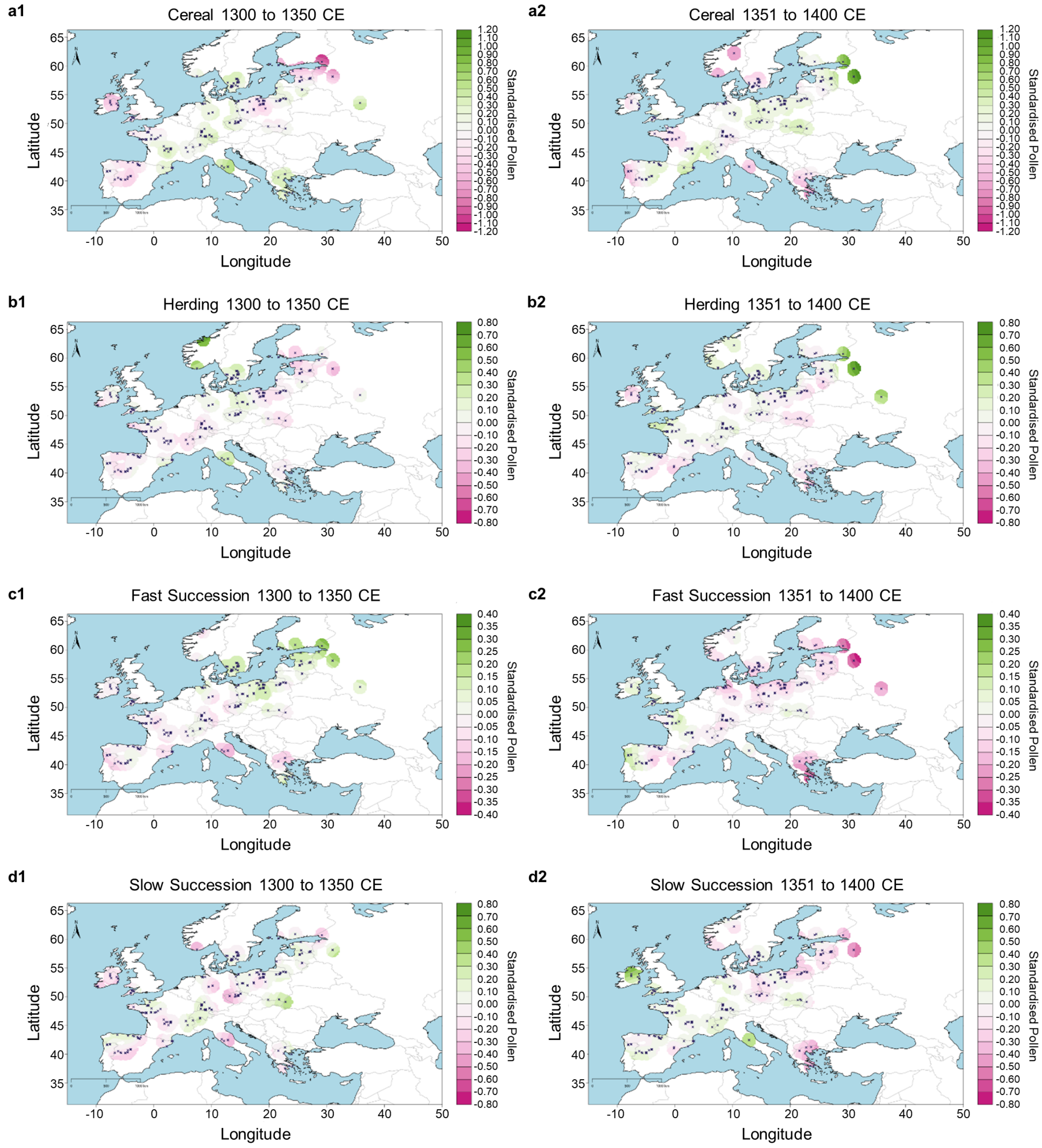

Supplementary Figure 5. Spatial extrapolation for all four indicators *c*. 50 years before and after the Black Death. **a**) Cereal 1301-1350 CE (1) and 1351-1400 CE (2) **b**) Herding 1301-1350 CE (1) and 1351-1400 CE (2) **c**) Fast Succession 1301-1350 CE (1) and 1351-1400 CE (2) **d**) Slow Succession 1301-1350 CE (1) and 1351-1400 CE (2).

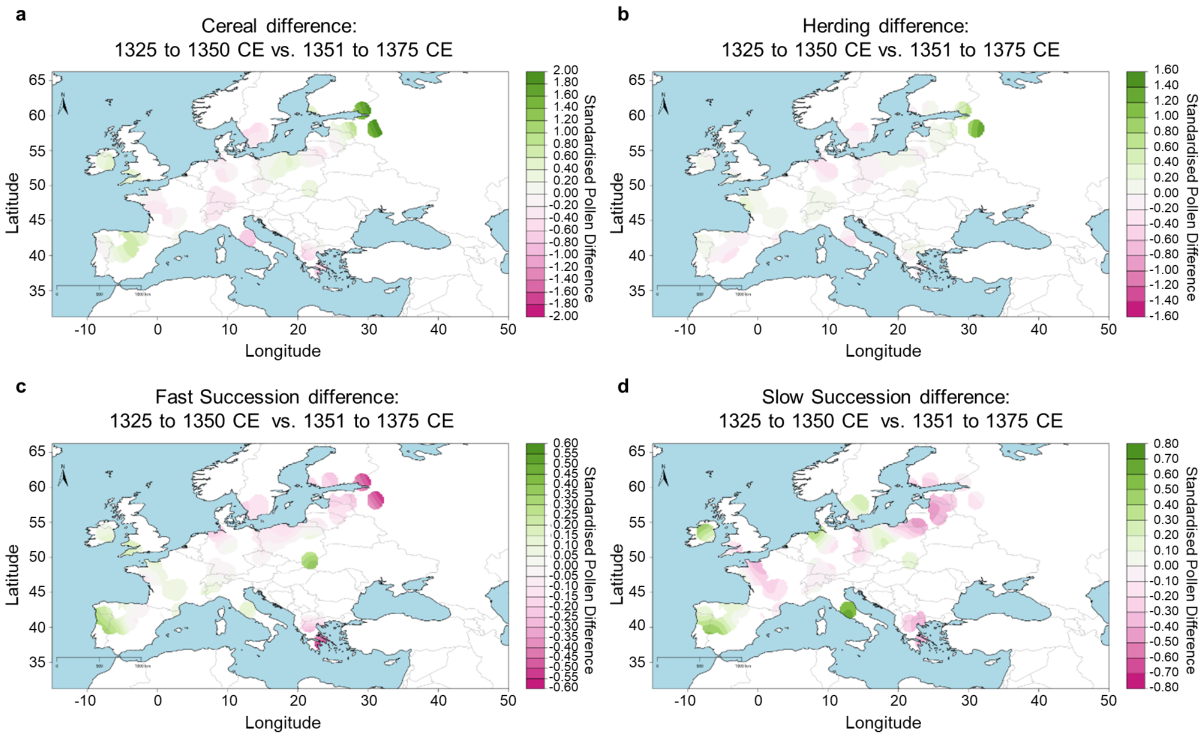

Supplementary Figure 6. ***Spatial extrapolation showing*** *1325-1350 vs 1351-1375* ***CE temporal variation in the BDP pollen indicators a****) Cereal* ***b****) Herding* ***c****) Fast Succession* ***d****) Slow Succession. Based on results from Supplementary Fig. 7.*

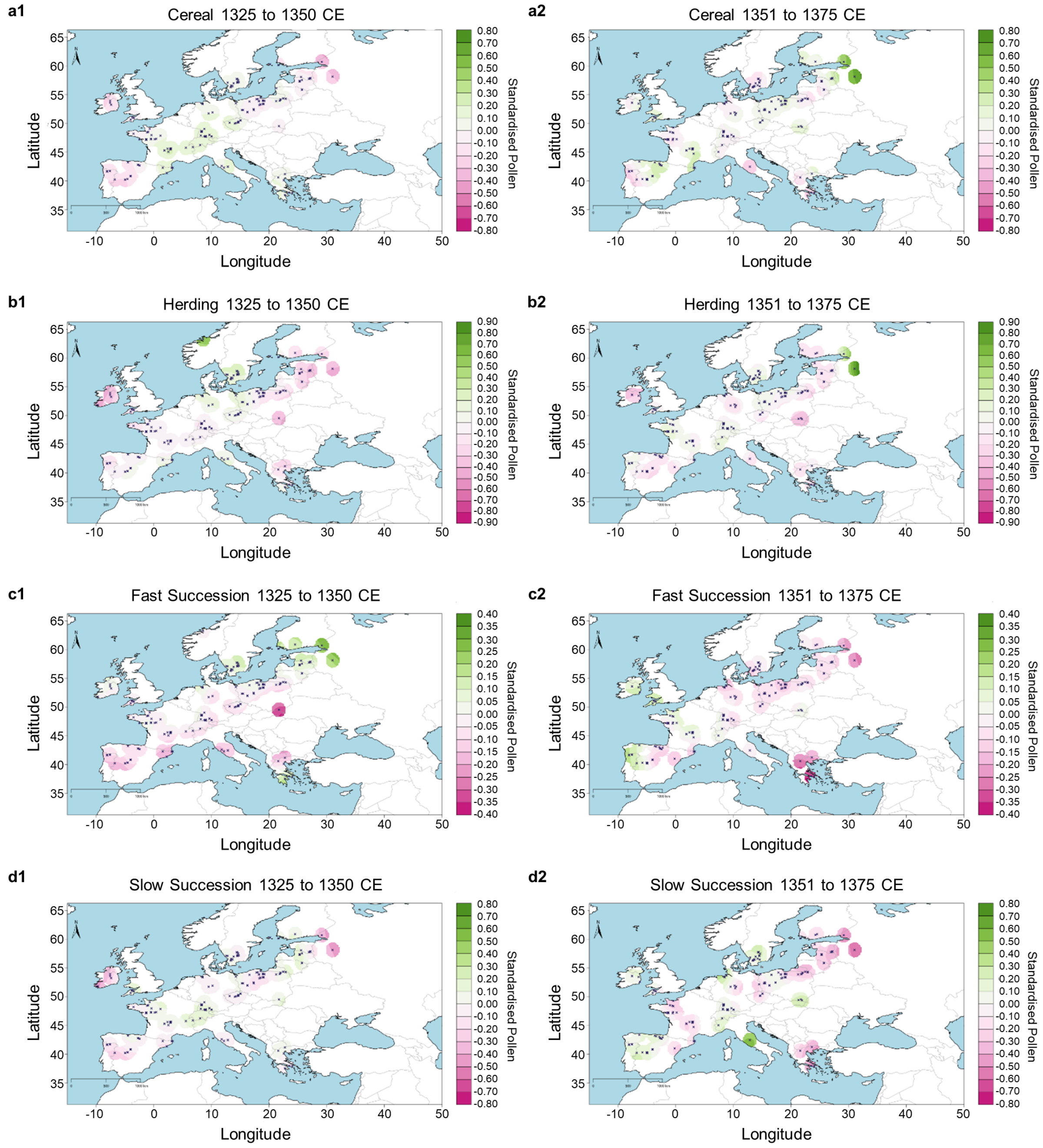

Supplementary Figure 7. Spatial extrapolation for all four indicators *c*. 25 years before and after the Black Death. **a**) Cereal 1325-1350 CE (1) and 1351-1375 CE (2) **b**) Herding 1325-1350 CE (1) and 1351-1375 CE (2) **c**) Fast Succession 1325-1350 CE (1) and 1351-1375 CE (2) **d**) Slow Succession 1325-1350 CE (1) and 1351-1375 CE (2).

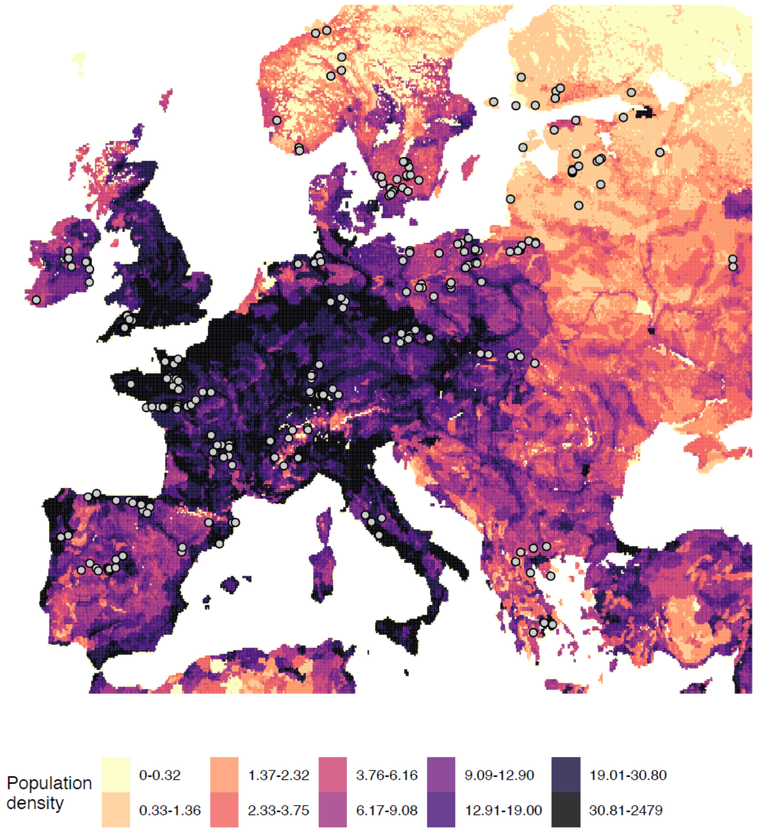

Supplementary Figure 8. Population density in Europe in ca 1300 CE and the location of pollen sites included in this study. Based on: HYDE version 3.2.1, Klein Goldewijk et al., Earth Syst. Sci. Data, 9, 927-953 (2017).

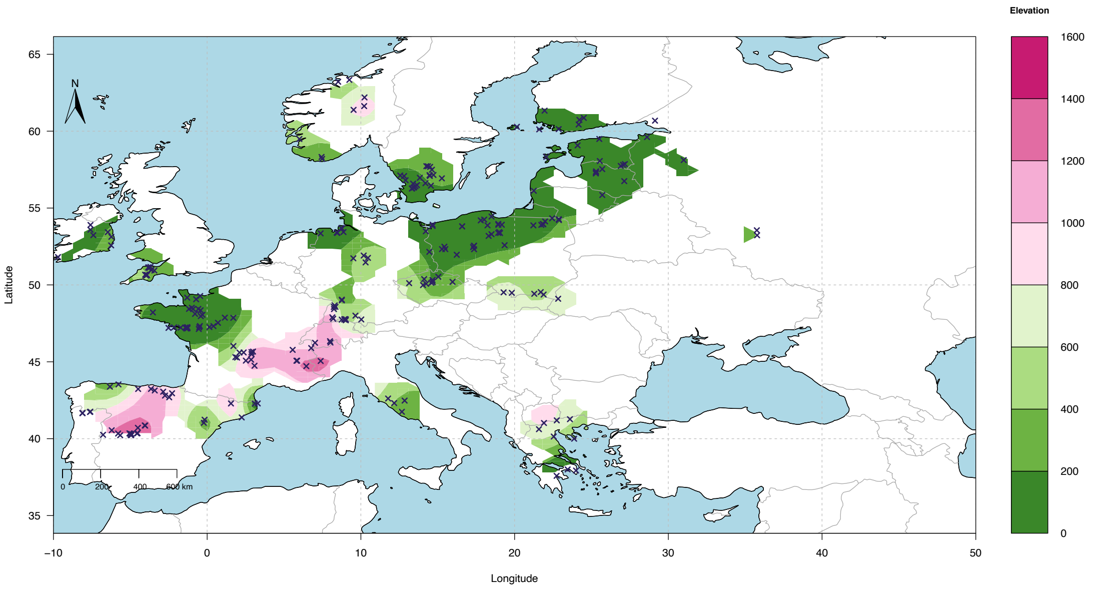

Supplementary Figure 9. Spatial extrapolation for site elevation, showing differences in average elevation for each region (smoothing settings set to a minimum). Based on information from Supplementary Data 1.
