# Supplementary material for: Palaeoecological data indicates land-use changes across Europe linked to spatial heterogeneity in mortality during the Black Death pandemic
Source: Nat Ecol Evol. 2022 Feb 10;6(3):297–306. doi: 10.1038/s41559-021-01652-4 (PMC8913360; doi:10.1038/s41559-021-01652-4)
Supplement: Supplementary file 2 — Reporting Summary. [file 41559_2021_1652_MOESM2_ESM.pdf]

nature portfolio

Corresponding author(s): Alessia Masi

Last updated by author(s): Oct 15, 2021

Reporting Summary

Nature Portfolio wishes to improve the reproducibility of the work that we publish. This form provides structure for consistency and transparency in reporting. For further information on Nature Portfolio policies, see our Editorial Policies and the Editorial Policy Checklist.

Statistics

For all statistical analyses, confirm that the following items are present in the figure legend, table legend, main text, or Methods section.

n/a	Confirmed	
☐	☒	The exact sample size (*n*) for each experimental group/condition, given as a discrete number and unit of measurement
☐	☒	A statement on whether measurements were taken from distinct samples or whether the same sample was measured repeatedly
☒	☐	The statistical test(s) used AND whether they are one- or two-sided<br>*Only common tests should be described solely by name; describe more complex techniques in the Methods section.*
☒	☐	A description of all covariates tested
☒	☐	A description of any assumptions or corrections, such as tests of normality and adjustment for multiple comparisons
☐	☒	A full description of the statistical parameters including central tendency (e.g. means) or other basic estimates (e.g. regression coefficient) AND variation (e.g. standard deviation) or associated estimates of uncertainty (e.g. confidence intervals)
☒	☐	For null hypothesis testing, the test statistic (e.g. *F*, *t*, *r*) with confidence intervals, effect sizes, degrees of freedom and *P* value noted<br>*Give P values as exact values whenever suitable.*
☒	☐	For Bayesian analysis, information on the choice of priors and Markov chain Monte Carlo settings
☒	☐	For hierarchical and complex designs, identification of the appropriate level for tests and full reporting of outcomes
☒	☐	Estimates of effect sizes (e.g. Cohen's *d*, Pearson's *r*), indicating how they were calculated

Our web collection on statistics for biologists contains articles on many of the points above.

Software and code

Policy information about availability of computer code

Data collection	No software was used to collect data.
Data analysis	Statistical analysis were performed in R 4.0.3. We have used libraries: sf, ggplot2, wBoot, dplyr.

For manuscripts utilizing custom algorithms or software that are central to the research but not yet described in published literature, software must be made available to editors and reviewers. We strongly encourage code deposition in a community repository (e.g. GitHub). See the Nature Portfolio guidelines for submitting code & software for further information.

Data

Policy information about availability of data

All manuscripts must include a data availability statement. This statement should provide the following information, where applicable:

- Accession codes, unique identifiers, or web links for publicly available datasets
- A description of any restrictions on data availability
- For clinical datasets or third party data, please ensure that the statement adheres to our policy

All data generated or analysed during this study are included in the published article (as the Supplementary Data).

Field-specific reporting

Please select the one below that is the best fit for your research. If you are not sure, read the appropriate sections before making your selection.

☐ Life sciences ☐ Behavioural & social sciences ☒ Ecological, evolutionary & environmental sciences

For a reference copy of the document with all sections, see nature.com/documents/nr-reporting-summary-flat.pdf

Ecological, evolutionary & environmental sciences study design

All studies must disclose on these points even when the disclosure is negative.

Study description	The regional variation in the Black Death's impact in Europe has been investigated using a new approach, Big Data Palaeoecology (BDP), based on collecting and analyzing existing pollen sequences from 19 European countries (published as well as unpublished).
Research sample	In order to create our dataset, we used existing online palynological databases (the European Pollen Database, www.europeanpollendatabase.net, and the Czech Quaternary Palynological Database, https://botany.natur.cuni.cz/palycz/), as well as personal contacts of the study authors and a systematic publication search. In this way, we identified palynological sites in Europe that meet the necessary chronological and resolution quality criteria for the investigated period of time (1250-1450 CE).
Sampling strategy	Pollen sequence with sufficient resolution (average temporal resolution of 58 years) and 14C-age control (or varve chronology) have been selected and grouped according to regional clusters. Single isolated sites from outside these clusters have been excluded because they do not allow the application of quantitative analysis.
Data collection	Pollen percentages of selected taxa have been collected from the 261 pollen sequences included in the study.
Timing and spatial scale	Europe, 1250-1450 CE
Data exclusions	Pollen sequences with low resolution; no cereal pollen; insufficient chronological control; no accompanying sites in the same region.
Reproducibility	The analytical protocol for pollen extraction and identification is reported in the original publications. The Pollen Sum includes all the terrestrial taxa with some exceptions based on the selection done in the original publications. The full list of sequences, exclusions from the Pollen Sum, age-depth models and full references are reported in Supplementary Data 1.
Randomization	The sequences are grouped in regions according to spatial distribution
Blinding	n/a

Did the study involve field work? ☐ Yes ☒ No

Reporting for specific materials, systems and methods

We require information from authors about some types of materials, experimental systems and methods used in many studies. Here, indicate whether each material, system or method listed is relevant to your study. If you are not sure if a list item applies to your research, read the appropriate section before selecting a response.

Materials & experimental systems

n/a	Involved in the study
☒	☐ Antibodies
☒	☐ Eukaryotic cell lines
☒	☐ Palaeontology and archaeology
☒	☐ Animals and other organisms
☒	☐ Human research participants
☒	☐ Clinical data
☒	☐ Dual use research of concern

Methods

n/a	Involved in the study
☒	☐ ChIP-seq
☒	☐ Flow cytometry
☒	☐ MRI-based neuroimaging
