# Supplementary material for: Palaeoecological data indicates land-use changes across Europe linked to spatial heterogeneity in mortality during the Black Death pandemic
Source: Nat Ecol Evol. 2022 Feb 10;6(3):297–306. doi: 10.1038/s41559-021-01652-4 (PMC8913360; doi:10.1038/s41559-021-01652-4)
Supplement: Supplementary file 3 — Peer Review Information. [file 41559_2021_1652_MOESM3_ESM.pdf]

natureresearch

Peer Review Information

Journal: Nature Ecology & Evolution
Manuscript Title: Palaeoecological data indicates land-use changes across Europe linked to spatial heterogeneity in mortality during the Black Death pandemic
Corresponding author name(s): Alessia Masi

Reviewer Comments & Decisions:

Decision Letter, initial version:

2nd August 2021

*Please ensure you delete the link to your author homepage in this e-mail if you wish to forward it to your co-authors.

Dear Dr Masi,

Your manuscript entitled "Big Data Palaeoecology reveals significant variation in Black Death mortality in Europe" has now been seen by four reviewers, whose comments are attached. The reviewers have raised a number of concerns which will need to be addressed before we can offer publication in Nature Ecology & Evolution. We will therefore need to see your responses to the criticisms raised and to some editorial concerns, along with a revised manuscript, before we can reach a final decision regarding publication.

In particular, you will see that the reviewers emphasise the need to tone down the degree to which the narrative presented is a surprise from a historical perspective--I don't think this compromises novelty since as reviewer 1 argues, it is valuable to show the palaeoecological dimension on land use change during the period. And as reviewer 4 comments, there is also a need to shore up the linkage between mortality and land use changes--bringing more historical perspective in here could help.

We therefore invite you to revise your manuscript taking into account all reviewer and editor comments. Please highlight all changes in the manuscript text file [OPTIONAL: in Microsoft Word format].

We are committed to providing a fair and constructive peer-review process. Do not hesitate to contact us if there are specific requests from the reviewers that you believe are technically impossible or unlikely to yield a meaningful outcome.

When revising your manuscript:

* Include a “Response to reviewers” document detailing, point-by-point, how you addressed each reviewer comment. If no action was taken to address a point, you must provide a compelling argument. This response will be sent back to the reviewers along with the revised manuscript.

* If you have not done so already please begin to revise your manuscript so that it conforms to our Article format instructions at http://www.nature.com/natecolevol/info/final-submission. Refer also to any guidelines provided in this letter.

* Include a revised version of any required reporting checklist. It will be available to referees (and, potentially, statisticians) to aid in their evaluation if the manuscript goes back for peer review. A revised checklist is essential for re-review of the paper.

Please use the link below to submit your revised manuscript and related files:

[REDACTED]

Note: This URL links to your confidential home page and associated information about manuscripts you may have submitted, or that you are reviewing for us. If you wish to forward this email to co-authors, please delete the link to your homepage.

We hope to receive your revised manuscript within four to eight weeks. If you cannot send it within this time, please let us know. We will be happy to consider your revision so long as nothing similar has been accepted for publication at Nature Ecology & Evolution or published elsewhere.

Nature Ecology & Evolution is committed to improving transparency in authorship. As part of our efforts in this direction, we are now requesting that all authors identified as ‘corresponding author’ on published papers create and link their Open Researcher and Contributor Identifier (ORCID) with their account on the Manuscript Tracking System (MTS), prior to acceptance. ORCID helps the scientific community achieve unambiguous attribution of all scholarly contributions. You can create and link your ORCID from the home page of the MTS by clicking on ‘Modify my Springer Nature account’. For more information please visit please visit www.springernature.com/orcid.

Please do not hesitate to contact me if you have any questions or would like to discuss these revisions further.

We look forward to seeing the revised manuscript and thank you for the opportunity to review your work.

[REDACTED]

Reviewer expertise:

Reviewer #1: medieval economic history

Reviewer #2: Black Death archaeology

Reviewer #3: palaeoecology and palynology

Reviewer #4: palaeoecology and palynology

Reviewers' comments:

Reviewer #1 (Remarks to the Author):

This is an interesting article about the regional variation of agrarian changes in Europe during the late middle ages. Probably for the first time such a large amount of data based on pollen analysis has been brought together to study the regional variation and changes of rural cultivation activity during that period.

It confirms the existing knowledge (already since Biraben's publication of 1975 and many others after that) that the influence of the late medieval crisis hit Europe with a huge regional variation. However one of the most important merits of the article is that it is the first time that a study like this is done on the basis a large scale dataset out of the positive sciences.

However, contrary to what the title and some parts of the article are suggesting, the data do not exclusively reveal the impact of the pest epidemic of 1347-1352. They are on the contrary revealing also other changes in the late medieval society that are due to other factors as well. Indeed other demographic catastrophes were typical for the (later) middle ages as well and certainly also are reflected in pollen analyses. We think about famines (e.g. a° 1315-1317), wars (that became much more devastating in the late middle ages and also caused epidemic diseases) but especially other epidemics and most importantly the s.c. 'echo-(pest-)epidemics' after the first plague epidemic (e.g. 1357-9, 1373, 1400-1401…). However also these epidemics occurred with huge regional variations. Moreover during that period structural changes of the rural society took place that were only partly linked to the black death (e.g. land concentration and the occurrence of new elites).

To conclude: Interesting article but before publication we advise that the content should be changed as mentioned and that a new title should be formulated that is more conform to its content.

Reviewer #2 (Remarks to the Author):

This interesting study uses changes in the type of pollen in sediment cores to investigate the consequences of the black death upon the types of plant growing. The study uses a logical approach, and the illustrations explain the data convincingly.

Estimating Black Death Mortality section (lines 131-165). You write that until now texts have been the best way to study the impact of the Black Death on population size, although it is good that you do mention papers about changing pollution levels. However, other techniques are available. One study looked at the number of fragments of pottery in test pits to show that there was a significant drop in

pottery use and production in England following the Black Death, likely a result of the smaller population size. I would encourage you to read this and give a couple of sentences on its approach (it is not my paper):
Lewis, C. (2016) Disaster recovery: new archaeological evidence for the long-term impact of the 'calamitous' fourteenth century. Antiquity 90(351): 777-797.

Minor point:
Abstract first sentence: 'reknown' should read 'renown'.

Reviewer #3 (Remarks to the Author):

This is a very interesting and topical paper. In principle, I was surprised by the methodological approach, but after studying it repeatedly, I find that the idea is provocative and has great explanatory power. The conclusion about the spatial and social heterogeneity of the epidemic is common sense regardless of conventional historicist approaches based on inevitably biased sources.

Obviously, pollen analysis also entails its spatial sampling, analysis and interpretation biases, in addition to the biases derived from the resolution in the identification of palynological types. Not even close to all the pollen analyses published in the studied territory are presented here either. Naturally, all these limitations are unapproachable in the current state of collaboration with Big Data.

That said, the epistemological and methodological approach of this study seems fresh and healthy to me as a pioneering study on the frontiers of knowledge. A much needed consilience between the natural sciences and the social sciences. A brilliant work in my opinion, which will be a must-read for the general public and which I predict a considerable media success.

My enthusiasm cannot but lead to the belief that the paper can be published in its current form. The language is accessible, the information well collected and the message clear and direct, without frills. Congratulations to the authors.

Reviewer #4 (Remarks to the Author):

In "Big Data Palaeoecology reveals significant variation in Black Death mortality in Europe" the authors evaluate paleorecord of series of sedimentary sequences from 19 European countries to evaluate whether landscape changes could be linked with the hypothesis that half of the population died within a single year. This is a very interesting paper with an interesting approach and I have enjoyed reading it. It is really exciting seeing how fossil pollen time-series are answering big and relevant questions of concern to society as pandemics are. However, I have some concerns I would like the authors to consider.

Main points:

1) Mortality and landscape change: After reading the paper with interest and SI (lines 445 474) the

“direct” link between population decline or mortality and landscape change that I think is the main hypothesis of the paper is not clear to me. The authors used >1600 palynological time-series to evaluate the demographic impact of the Black Death on a regional scale across Europe. I think that the results of the paper are not strictly quantifying mortality (e.g. what magnitude?) but responses of local/regional vegetation/landscapes to changes on land-use, and other factors including mortality. However, I think the results are still relevant.

I also found that the manuscript requires an improved discussion on demographic changes-how the authors interpret demographic changes in their results? It is mortality quantified “only” with th increase and decrease of cereals, etc? In Lines 191-198 there are more discussion on validation. And I thank the authors for including this but I keep struggling in understanding what is the actual quantification of mortality. Also, why Poland experienced growth? More is required in here.

2) Time period: It requires more detail on why the analysis are focusing on the period between 1250 and 1450 CE. I think this detail should be explored in-depth to better discuss the otherwise relevant results.

3) Regional selection: Another issues I think it requires more description/discussion is why the authors included and excluded sites (Fig 1). I understand the limitation on finding good quality datasets for the time period of interest but I think that comparing sites located in mountain areas vs coastal areas and/or with different population densities, etc. probably impacted the findings.

Minor points

Line 113: I think it is missing the years that the pandemic was active. Please add the years.

Ecological indices: It is missing a line with the relevance of these indices to help linking landscape change and the scale of the Black Death mortality. Why are these indices important to answer the question? I read the detail in SI but still not clear to me why the authors are using them. Not suggesting that the approach is not relevant but there are so many analysis to quantify how cereals and different vegetation types change over time that it require a better justification.

Lines 171-172: The authors suggested that “Pollen data can be used to assess past demographic dynamics as human pressure on the landscape in the preindustrial period was directly dependent on the availability of rural labour.” I think this line is a bit confusing as it is mixing up to rural vs city demographics with the main point of the paper that is mortality.

Line 261: This is a very strong sentence “The significant variability of Black Death mortality that our BDP approach identifies remains...” I’m struggling to accept that the results are showing differences in mortality rates. I think that it is an interesting hypothesis but it could well be that the landscape change found may respond to differences in vegetation resilience, different baseline population densities and cultures, land-use, culture differences, etc.

Lines 286-295 probably could be placed at the beginning of the manuscript to help integrating the spatial distribution of the Black Death and to better justify the time-period.

Lines 487-494: Depth age models- have the authors re-calibrated them using the new calibration curves?

I hope my comments are helpful to improve the manuscript.

*******************END*******************

Author Rebuttal to Initial comments

Response to reviewers

Big Data Palaeoecology reveals significant variation in Black Death mortality in Europe

Izdebski et al.

Editor: In particular, you will see that the reviewers emphasise the need to tone down the degree to which the narrative presented is a surprise from a historical perspective--I don't think this compromises novelty since as reviewer 1 argues, it is valuable to show the palaeoecological dimension on land use change during the period.

-> We toned down the first section of the article and took into account the non-Anglophone historiography, which contrary to the recent English-speaking consensus on the Black Death as a universal catastrophic event, noticed some regional variation during the initial phases of the second plague pandemic.

And as reviewer 4 comments, there is also a need to shore up the linkage between mortality and land use changes--bringing more historical perspective in here could help.

-> We rewrote the relevant (first) section of the Methods, providing it with additional references. In particular, we divided this section into two clearly distinguished main points: (1) the close connection between landscape changes as revealed by palynology and population dynamics as revealed by historical sources, as proved by several recent historical-palynological studies; (2)

the landscape changes to be expected in plague-struck areas during the Black Death, based on the current consensus on estimated population decline among the historians of the Black Death.

Reviewer expertise:

Reviewer #1: medieval economic history

Reviewer #2: Black Death archaeology

Reviewer #3: palaeoecology and palynology

Reviewer #4: palaeoecology and palynology

Reviewer #1 (Remarks to the Author):

This is an interesting article about the regional variation of agrarian changes in Europe during the late middle ages. Probably for the first time such a large amount of data based on pollen analysis has been brought together to study the regional variation and changes of rural cultivation activity during that period.

It confirms the existing knowledge (already since Biraben's publication of 1975 and many others after that) that the influence of the late medieval crisis hit Europe with a huge regional variation. However one of the most important merits of the article is that it is the first time that a study like this is done on the basis a large scale dataset out of the positive sciences.

However, contrary to what the title and some parts of the article are suggesting, the data do not exclusively reveal the impact of the pest epidemic of 1347-1352. They are on the contrary revealing also other changes in the late medieval society that are due to other factors as well. Indeed other demographic catastrophes were typical for the (later) middle ages as well and certainly also are reflected in pollen analyses. We think about famines (e.g. a° 1315-1317), wars (that became much more devastating in the late middle ages and also caused epidemic

diseases) but especially other epidemics and most importantly the s.c. ‘echo-(pest-)epidemics’ after the first plague epidemic (e.g. 1357-9, 1373, 1400-1401…). However also these epidemics occurred with huge regional variations. Moreover during that period structural changes of the rural society took place that were only partly linked to the black death (e.g. land concentration and the occurrence of new elites).

-> We are grateful for these careful and constructive comments. We had long discussions among the historians who co-authored this paper about the extent to which our results are connected with phenomena other than the Black Death itself. Our conclusion is that our method produces results that actually are connected directly to the Black Death understood as the initial introduction of *Y. pestis* to Europe during the second pandemic. Of course, the later echo-epidemics (later outbreaks) amplified the consequences of the Black Death, but the role of the outbreak of the Black Death was crucial for whether a population collapse - leading to significant landscape changes - occurred in a particular region or not. It is the Black Death itself, the pandemic of the mid-fourteenth-century, that is attributed with killing half of the population of Europe in a few years, not the subsequent outbreaks, which only added, albeit in a regionalized way, to the mortality of the Black Death decades after the Black Death. As such, it is the Black Death that we focus on. It was the major demographic moment of change in fourteenth-century European history and, in fact, in late medieval history more generally. Very few historians would expect the landscape of a region to have been altered significantly if it was not struck by the Black Death, but only by secondary and tertiary outbreaks of plague after the Black Death in the fourteenth century.

To these remark, we should add that our method is not focused on showing the different trajectories of the late medieval crisis (as was, for instance, Izdebski et al. 2016, https://doi.org/10.1016/j.eeh.2015.10.003); instead of focusing on time series and trend analysis, we focus on the extent to which a single event, well-constrained in time - the Black Death - can be associated with landscape changes across Europe. It may well be that all the other phenomena that are collectively known as the late medieval crisis left their imprint on the landscape as well (for instance, our data show the impact of the Great Famine of 1315-1317 in Devon/England, for which we have no significant impact of the Black Death), but our method in this particular article is set up in such a way that it does not reveal them: it is very clearly focused on the Black Death.

Hence, the results presented in our article concern 100-year periods before and after the Black Death, that is 4 human generations (the minimum time to overcome a sudden population loss in the range of 30-50%). Additionally, in the Supplementary Material, we already showed the results for 50 and 25 year periods before and after the Black Death: they

are very similar to the results of the 100 year periods, as we discussed in the main text. To emphasise this fact, and further demonstrate the focus of our analysis on the Black Death in particular, we now added additional figures to the text and commented on them. Based on the consistency between these different periods of analysis, we are convinced that the landscape changes we analyse across Europe are related directly to the Black Death, and not - as the reviewer suggests - to the 'Dantean Anomaly' and the Great Famine of the 1310s or to the late fourteenth or early fifteenth century-plague outbreaks. The impact of these phenomena is not captured by the 25 year or even 50 year periods of analysis). Thus, given the current consensus hypothesis we want to test (universal mass mortality), we are able to demonstrate that whereas it did occur in some areas (e.g. Greece, Sweden or Italy), it did not occur elsewhere (e.g. Poland or Spain). We believe this is the main merit of our article, and to some extent it is indeed confirmation of historical works of Biraben, Małowist or Gottfried, who emphasised regional differentiation of the late medieval crisis in Europe, a minority perspective today which we now make clear reference to in the article).

To conclude: Interesting article but before publication we advise that the content should be changed as mentioned and that a new title should be formulated that is more conform to its content.

-> We have suggested a new title, clearly focused on the vision of the Black Death that dominates plague studies and the public imagination - the vision which we falsify in our study.

Reviewer #2 (Remarks to the Author):

This interesting study uses changes in the type of pollen in sediment cores to investigate the consequences of the black death upon the types of plant growing. The study uses a logical approach, and the illustrations explain the data convincingly.

Estimating Black Death Mortality section (lines 131-165). You write that until now texts have been the best way to study the impact of the Black Death on population size, although it is good that you do mention papers about changing pollution levels. However, other techniques are available. One study looked at the number of fragments of pottery in test pits to show that there was a significant drop in pottery use and production in England following the Black Death, likely a result of the smaller population size. I would encourage you to read this and give a couple of sentences on its approach (it is not my paper):
Lewis, C. (2016) Disaster recovery: new archaeological evidence for the long-term impact of the 'calamitous' fourteenth century. Antiquity 90(351): 777-797.

-> We have now included this study in our method section. We also would like to thank the reviewer 2 for the positive comments on our paper and for expanding even further our interdisciplinarity.

Minor point:
Abstract first sentence: ‘reknown’ should read ‘renown’.

-> Corrected, thank you.

Reviewer #3 (Remarks to the Author):

This is a very interesting and topical paper. In principle, I was surprised by the methodological approach, but after studying it repeatedly, I find that the idea is provocative and has great explanatory power. The conclusion about the spatial and social heterogeneity of the epidemic is common sense regardless of conventional historicist approaches based on inevitably biased sources.

Obviously, pollen analysis also entails its spatial sampling, analysis and interpretation biases, in addition to the biases derived from the resolution in the identification of palynological types. Not even close to all the pollen analyses published in the studied territory are presented here either. Naturally, all these limitations are unapproachable in the current state of collaboration with Big Data.

That said, the epistemological and methodological approach of this study seems fresh and healthy to me as a pioneering study on the frontiers of knowledge. A much needed consilience between the natural sciences and the social sciences. A brilliant work in my opinion, which will be a must-read for the general public and which I predict a considerable media success.

My enthusiasm cannot but lead to the belief that the paper can be published in its current form. The language is accessible, the information well collected and the message clear and direct, without frills. Congratulations to the authors.

-> Thank you very much for such an encouraging review!

Reviewer #4 (Remarks to the Author):

In “Big Data Palaeoecology reveals significant variation in Black Death mortality in Europe” the authors evaluate paleorecord of series of sedimentary sequences from 19 European countries to evaluate whether landscape changes could be linked with the hypothesis that half of the population died within a single year. This is a very interesting paper with an interesting approach and I have enjoyed reading it. It is really exciting seeing how fossil pollen time-series are answering big and relevant questions of concern to society as pandemics are. However, I have some concerns I would like the authors to consider.

Main points:

1) Mortality and landscape change: After reading the paper with interest and SI (lines 445 474) the “direct” link between population decline or mortality and landscape change that I think is the main hypothesis of the paper is not clear to me. The authors used >1600 palynological time-series to evaluate the demographic impact of the Black Death on a regional scale across Europe. I think that the results of the paper are not strictly quantifying mortality (e.g. what magnitude?) but responses of local/regional vegetation/landscapes to changes on land-use, and other factors including mortality. However, I think the results are still relevant.

-> Yes, indeed. In agreement with the comments of R1, and by changing the title, we now tried to make clear that while we do study landscape changes (or lack thereof) , we do so entirely in order to test the currently dominant view that the Black Death led to universal mortality within the range of 30-50%. We now make clear in the methods section why this is important to us: such a truly significant mortality would lead to profound and sudden landscape change, which we indeed observe in some of our study regions.

I also found that the manuscript requires an improved discussion on demographic changes-how the authors interpret demographic changes in their results? It is mortality quantified “only” with th increase and decrease of cereals, etc? In Lines 191-198 there are more discussion on validation. And I thank the authors for including this but I keep struggling in understanding what is the actual quantification of mortality.

-> Entirely understood. We have now improved our discussion of the connection to plague mortality in the relevant sections of the Methods section and in the main text. As we explain, we do not provide direct, concrete estimates of mortality, as the state of the research on palynology and historical demography has not yet reached this stage, despite some major

recent advances in this respect. Our aim instead is to use pollen data to verify the popular vision of the Black Death as a universal mortality catastrophe in Europe, leading to 30-50% of the population dying in a few mere years. As we now try to explain more clearly, such a scenario would result in major landscape changes following the sudden and profound reduction of human pressure on the landscape. We observe significant population erosion in some parts of Europe (consistently with the limited written evidence available for Black Death mortality), while not in others (for which we provide explanation in the concluding section of the main text).

Also, why Poland experienced growth? More is required in here.

-> We now explain this fact.

2) Time period: It requires more detail on why the analysis are focusing on the period between 1250 and 1450 CE. I think this detail should be explored in-depth to better discuss the otherwise relevant results.

-> We now discuss our choice of 100 year slices of time on either side of 1350 as our main period of analysis in the first section of the Methods. We now also discuss the results we obtained for shorter time periods in the main text.

3) Regional selection: Another issues I think it requires more description/discussion is why the authors included and excluded sites (Fig 1). I understand the limitation on finding good quality datasets for the time period of interest but I think that comparing sites located in mountain areas vs coastal areas and/or with different population densities, etc. probably impacted the findings.

-> We now provide two additional supplementary figures that show the geographical setting (lowland vs highland) of our sites as well as the population densities around our sites in the fourteenth century. These variables are not linked to the results we obtained for our BDP pollen indicators, however. Please note that we undertook as well a comparative study of change in the landscape over periods of 200, 100 and 50 years. We assume that over such short time scales physical geographical characteristics of each site remain stable and do not impact our results and conclusions.

Minor points

Line 113: I think it is missing the years that the pandemic was active. Please add the years.

-> This Black Death pandemic probably began in the 1330s in Central Asia and in many parts of Eurasia and Africa - outside of Europe - continued until the late 1350s. For this reason, we believe providing an approximate chronological range of the “mid-14th c.” is most correct. For the entire Eurasia and Africa, providing set years would be difficult to substantiate and there is no consensus (contrary to the dates for Europe only).

Ecological indices: It is missing a line with the relevance of these indices to help linking landscape change and the scale of the Black Death mortality. Why are these indices important to answer the question? I read the detail in SI but still not clear to me why the authors are using them. Not suggesting that the approach is not relevant but there are so many analysis to quantify how cereals and different vegetation types change over time that it require a better justification.

-> We have now provided more explanation in the main text and in the Methods.

Lines 171-172: The authors suggested that “Pollen data can be used to assess past demographic dynamics as human pressure on the landscape in the preindustrial period was directly dependent on the availability of rural labour.” I think this line is a bit confusing as it is mixing up to rural vs city demographics with the main point of the paper that is mortality.

-> As we explain in the Methods, ~90% of the late medieval European population lived in the countryside, so in order to understand overall mortality levels during the Black Death, our primary focus should be the rural populations.

Line 261: This is a very strong sentence “The significant variability of Black Death mortality that our BDP approach identifies remains...” I’m struggling to accept that the results are showing differences in mortality rates. I think that it is an interesting hypothesis but it could well be that

the landscape change found may respond to differences in vegetation resilience, different baseline population densities and cultures, land-use, culture differences, etc.

-> Duly noted. As we argue in the Methods, though, the changes we observe are related to human pressure on the landscape, which in turn was dependent on population levels. We also focus primarily on cultivated plants, cereals, whose presence in the landscape diminishes dramatically and over very short time scales without human activity/pressure that supports these plants, as we explain in the Methods. Moreover, thanks to our strict chronological focus, also as now explained further in response to the comments of R1, we are in the position to argue that these landscape changes (or lack thereof) were directly related to the Black Death, that is, the commonly supposed most important demographic event of the fourteenth century. At the same time, we agree with R4 that the factors s/he mentions were important as moderating the Black Death's impact at the regional and local level - and we discuss this point in the concluding section of the main text.

Lines 286-295 probably could be placed at the beginning of the manuscript to help integrating the spatial distribution of the Black Death and to better justify the time-period.

-> We now focus more on explaining the temporal and spatial focus we have adopted throughout the text.

Lines 487-494: Depth age models- have the authors re-calibrated them using the new calibration curves?

-> Yes, absolutely: as we explain in the Methods, we re-calculated age-depth models for a large portion of our sites: especially for those that were using the older calibration curves.

I hope my comments are helpful to improve the manuscript.

-> Yes, indeed, thank you very much!

naturereserch

Decision Letter, first revision:

8th October 2021

Dear Dr. Masi,

Thank you for submitting your revised manuscript "The Black Death Killed Far Fewer Europeans than Commonly Thought, Big Data Paleoecology Reveals" (NATECOLEVOL-210614034A). It has now been seen again by the original reviewers and their comments are below. The reviewers find that the paper has improved in revision, and therefore we'll be happy in principle to publish it in Nature Ecology & Evolution, pending revisions to satisfy the reviewers' final requests and to comply with our editorial and formatting guidelines. As explained below, our full recommendations will be sent in a later email, but for now, please note that both reviewers recommend that considerable nuance be brought into the discussion--your main text is on the short side by my rough calculation, so I estimate that you should be able to incorporate up to about 500 words of additional discussion which should address the remaining reviewer concerns.

If the current version of your manuscript is in a PDF format, please email us a copy of the file in an editable format (Microsoft Word or LaTex)-- we can not proceed with PDFs at this stage.

We are now performing detailed checks on your paper and will send you a checklist detailing our editorial and formatting requirements in about a week. Please do not upload the final materials and make any revisions until you receive this additional information from us.

Thank you again for your interest in Nature Ecology & Evolution. Please do not hesitate to contact me if you have any questions.

[REDACTED]

Reviewer #1 (Remarks to the Author):

GENERAL COMMENT:
The article has been upgraded a lot: the amount of relevant literature has been increased and the text is more nuanced.
A few remarks anyway (put in the reply-text of the authors)

DETAILS:
Reply text of the authors to the first remarks:
"Very few historians would expect the landscape of a region to have been altered significantly if it was not struck by the Black Death, but only by secondary and tertiary outbreaks of plague after the Black Death in the fourteenth century. "
Reply of the reviewer: Anyway there were important regions where the later outbreaks had much more influence that the first outbreak. Moreover if there would not have been 'echo-epidemics' , the economy (and the landscapes) probably would

have recovered early.

Reply text of the authors to the review
“To these remark, we should add that our method is not focused on showing the different trajectories of the late medieval crisis (as was, for instance, Izdebski et al. 2016, https://doi.org/10.1016/j.eeh.2015.10.003); instead of focusing on time series and trend analysis, we focus on the extent to which a single event, well constrained in time - the Black Death - can be associated with landscape changes across Europe. It may well be that all the other phenomena that are collectively known as the late medieval crisis left their imprint on the landscape as well (for instance, our data show the impact of the Great Famine of 1315-1317 in Devon/England, for which we have no significant impact of the Black Death), but our method in this particular article is set up in such a way that it does not reveal them: it is very clearly focused on the Black Death.”

Reply of the reviewer:
My remark remains...Why should the data reveal more the influence of the black dead than other elements of the rural economy that were changing in the later middle ages? Of course it is logic that one cannot deal with these other elements but at least it could be mentioned.

Reviewer #4 (Remarks to the Author):

This version of the manuscript reads very well and I thank the authors for taking into consideration my comments. This paper is interesting, well written, with fantastic figures, and can bring good discussion on how to integrate data from different paleo-sciences. I agree that population changes may result in major landscape changes (accounted using pollen) following the sudden and profound reduction of human pressure on the landscape that the BD produced-we have a good example with COVID-19. However, I keep struggling with the concept that the land-use changes found are reflecting differences in mortality rates or the magnitude of the severity of BD, only (line 313, title, etc).

Overall, the authors should tone down a bit more the narrative around novelty (e.g. pioneering). Using pollen data (or Big-data palaeoecology as the authors refers too) to infer land-use changes or landscape changes is not novel as it is not the link between land-use change and population dynamics. What is novel and exciting of this paper is the integration of historical and paleoecological sources to answer a current important research question: what happen to the landscape when there is a global pandemic with all the associated factors (economical crisis, rates of mortality, duration of the pandemic, type of vegetation, etc…)?

Interesting paper!

natureresearch

13th October 2021

Dear Dr. Masi,

Thank you for your patience as we’ve prepared the guidelines for final submission of your Nature Ecology & Evolution manuscript, "The Black Death Killed Far Fewer Europeans than Commonly Thought, Big Data Paleoecology Reveals" (NATECOLEVOL-210614034A). Please carefully follow the step-by-step instructions provided in the attached file, and add a response in each row of the table to indicate the changes that you have made. Please also check and comment on any additional marked-up edits we have proposed within the text. Ensuring that each point is addressed will help to ensure that your revised manuscript can be swiftly handed over to our production team.

We would like to start working on your revised paper, with all of the requested files and forms, as soon as possible (preferably within two weeks). Please get in contact with us immediately if you anticipate it taking more than two weeks to submit these revised files.

When you upload your final materials, please include a point-by-point response to any remaining reviewer comments.

If you have not done so already, please alert us to any related manuscripts from your group that are under consideration or in press at other journals, or are being written up for submission to other journals (see: https://www.nature.com/nature-research/editorial-policies/plagiarism#policy-on-duplicate-publication for details).

In recognition of the time and expertise our reviewers provide to Nature Ecology & Evolution’s editorial process, we would like to formally acknowledge their contribution to the external peer review of your manuscript entitled "The Black Death Killed Far Fewer Europeans than Commonly Thought, Big Data Paleoecology Reveals". For those reviewers who give their assent, we will be publishing their names alongside the published article.

Nature Ecology & Evolution offers a Transparent Peer Review option for new original research manuscripts submitted after December 1st, 2019. As part of this initiative, we encourage our authors to support increased transparency into the peer review process by agreeing to have the reviewer comments, author rebuttal letters, and editorial decision letters published as a Supplementary item. When you submit your final files please clearly state in your cover letter whether or not you would like to participate in this initiative. Please note that failure to state your preference will result in delays in accepting your manuscript for publication.

Cover suggestions

As you prepare your final files we encourage you to consider whether you have any images or illustrations that may be appropriate for use on the cover of Nature Ecology & Evolution.

Covers should be both aesthetically appealing and scientifically relevant, and should be supplied at the best quality available. Due to the prominence of these images, we do not generally select images featuring faces, children, text, graphs, schematic drawings, or collages on our covers.

We accept TIFF, JPEG, PNG or PSD file formats (a layered PSD file would be ideal), and the image should be at least 300ppi resolution (preferably 600-1200 ppi), in CMYK colour mode.

If your image is selected, we may also use it on the journal website as a banner image, and may need to make artistic alterations to fit our journal style.

Please submit your suggestions, clearly labeled, along with your final files. We'll be in touch if more information is needed.

Nature Ecology & Evolution has now transitioned to a unified Rights Collection system which will allow our Author Services team to quickly and easily collect the rights and permissions required to publish your work. Approximately 10 days after your paper is formally accepted, you will receive an email in providing you with a link to complete the grant of rights. If your paper is eligible for Open Access, our Author Services team will also be in touch regarding any additional information that may be required to arrange payment for your article.

Please note that <i>Nature Ecology & Evolution</i> is a Transformative Journal (TJ). Authors may publish their research with us through the traditional subscription access route or make their paper immediately open access through payment of an article-processing charge (APC). Authors will not be required to make a final decision about access to their article until it has been accepted. Find out more about Transformative Journals

<B>Authors may need to take specific actions to achieve compliance with funder and institutional open access mandates. For submissions from January 2021, if your research is supported by a funder that requires immediate open access (e.g. according to Plan S principles) then you should select the gold OA route, and we will direct you to the compliant route where possible. For authors selecting the subscription publication route our standard licensing terms will need to be accepted, including our self-archiving policies. Those standard licensing terms will supersede any other terms that the author or any third party may assert apply to any version of the manuscript.

Please note that you will not receive your proofs until the publishing agreement has been received through our system.

For information regarding our different publishing models please see our Transformative Journals page. If you have any questions about costs, Open Access requirements, or our legal forms, please contact ASJournals@springernature.com.

Please use the following link for uploading these materials:

[REDACTED]

If you have any further questions, please feel free to contact me.

[REDACTED]

Reviewer #1:
Remarks to the Author:
GENERAL COMMENT:
The article has been upgraded a lot: the amount of relevant literature has been increased and the text is more nuanced.
A few remarks anyway (put in the reply-text of the authors)

DETAILS:
Reply text of the authors to the first remarks:
"Very few historians would expect the landscape of a region to have been altered significantly if it was not struck by the Black Death, but only by secondary and tertiary outbreaks of plague after the Black Death in the fourteenth century. "
Reply of the reviewer: Anyway there were important regions where the later outbreaks had much more influence that the first outbreak. Moreover if there would not have been 'echo-epidemics' , the economy (and the landscapes) probably would have recovered early.

Reply text of the authors to the review
"To these remark, we should add that our method is not focused on showing the different trajectories of the late medieval crisis (as was, for instance, Izdebski et al. 2016, https://doi.org/10.1016/j.eeh.2015.10.003); instead of focusing on time series and trend analysis, we focus on the extent to which a single event, well constrained in time - the Black Death - can be associated with landscape changes across Europe. It may well be that all the other phenomena that are collectively known as the late medieval crisis left their imprint on the landscape as well (for instance, our data show the impact of the Great Famine of 1315-1317 in Devon/England, for which we have no significant impact of the Black Death), but our method in this particular article is set up in such a way that it does not reveal them: it is very clearly focused on the Black Death."

Reply of the reviewer:
My remark remains...Why should the data reveal more the influence of the black dead than other elements of the rural economy that were changing in the later middle ages? Of course it is logic that one cannot deal with these other elements but at least it could be mentioned.

Reviewer #4:
Remarks to the Author:

This version of the manuscript reads very well and I thank the authors for taking into consideration my comments. This paper is interesting, well written, with fantastic figures, and can bring good discussion on how to integrate data from different paleo-sciences. I agree that population changes may result in major landscape changes (accounted using pollen) following the sudden and profound reduction of human pressure on the landscape that the BD produced-we have a good example with COVID-19. However, I keep struggling with the concept that the land-use changes found are reflecting differences in mortality rates or the magnitude of the severity of BD, only (line 313, title, etc).

Overall, the authors should tone down a bit more the narrative around novelty (e.g. pioneering). Using pollen data (or Big-data palaeoecology as the authors refers too) to infer land-use changes or landscape changes is not novel as it is not the link between land-use change and population dynamics. What is novel and exciting of this paper is the integration of historical and paleoecological sources to answer a current important research question: what happen to the landscape when there is a global pandemic with all the associated factors (economical crisis, rates of mortality, duration of the pandemic, type of vegetation, etc…)?

Interesting paper!

Author Rebuttal, first revision:

Response to the reviewers' comments (second revision)

Reviewer #1:
Remarks to the Author:
GENERAL COMMENT:
The article has been upgraded a lot: the amount of relevant literature has been increased and the text is more nuanced.

-> Thank you!

A few remarks anyway (put in the reply-text of the authors)

DETAILS:
Reply text of the authors to the first remarks:
"Very few historians would expect the landscape of a region to have been altered significantly if it was not struck by the Black Death, but only by secondary and tertiary outbreaks of plague after the Black Death in the fourteenth century. "
Reply of the reviewer: Anyway there were important regions where the later outbreaks had much more influence that the first outbreak. Moreover if there would not have been 'echo-

epidemics' , the economy (and the landscapes) probably would
have recovered early.

-> We took account of this point in the discussion.

Reply text of the authors to the review
"To these remark, we should add that our method is not focused on showing the different trajectories of the late medieval crisis (as was, for instance, Izdebski et al. 2016, https://doi.org/10.1016/j.eeh.2015.10.003); instead of focusing on time series and trend analysis, we focus on the extent to which a single event, well constrained in time - the Black Death - can be associated with landscape changes across Europe. It may well be that all the other phenomena that are collectively known as the late medieval crisis left their imprint on the landscape as well (for instance, our data show the impact of the Great Famine of 1315-1317 in Devon/England, for which we have no significant impact of the Black Death), but our method in this particular article is set up in such a way that it does not reveal them: it is very clearly focused on the Black Death."

Reply of the reviewer:
My remark remains...Why should the data reveal more the
influence of the black dead than other elements of the rural
economy that were changing in the later middle ages? Of
course it is logic that one cannot deal with these other
elements but at least it could be mentioned.

-> As suggested, we now discuss these elements.

Reviewer #4:
Remarks to the Author:
This version of the manuscript reads very well and I thank the authors for taking into consideration my comments. This paper is interesting, well written, with fantastic figures, and can bring good discussion on how to integrate data from different paleo-sciences. I agree that population changes may result in major landscape changes (accounted using pollen) following the sudden and profound reduction of human pressure on the landscape that the BD produced-we have a good example with COVID-19. However, I keep struggling with the concept that the land-use changes found are reflecting differences in mortality rates or the magnitude of the severity of BD, only (line 313, title, etc).

-> We modified the text in order to account for the context-dependent complexities involved in this potential link.

Overall, the authors should tone down a bit more the narrative around novelty (e.g. pioneering). Using pollen data (or Big-data palaeoecology as the authors refers too) to infer land-use changes or landscape changes is not novel as it is not the link between land-use change and population dynamics. What is novel and exciting of this paper is the integration of historical and paleoecological sources to answer a current important research question: what

happen to the landscape when there is a global pandemic with all the associated factors (economical crisis, rates of mortality, duration of the pandemic, type of vegetation, etc…)?

-> Thank you. We now try to make it clear – and the narrative has been toned down.

Interesting paper!

-> Thank you!

Final Decision Letter:

Dear Alessia,

We are pleased to inform you that your Article entitled "Palaeoecological data indicates land-use changes across Europe linked to spatial heterogeneity in mortality during the Black Death pandemic", has now been accepted for publication in Nature Ecology & Evolution.

Over the next few weeks, your paper will be copyedited to ensure that it conforms to Nature Ecology and Evolution style. Once your paper is typeset, you will receive an email with a link to choose the appropriate publishing options for your paper and our Author Services team will be in touch regarding any additional information that may be required

After the grant of rights is completed, you will receive a link to your electronic proof via email with a request to make any corrections within 48 hours. If, when you receive your proof, you cannot meet this deadline, please inform us at rjsproduction@springernature.com immediately.

You will not receive your proofs until the publishing agreement has been received through our system

Due to the importance of these deadlines, we ask you please us know now whether you will be difficult to contact over the next month. If this is the case, we ask you provide us with the contact information (email, phone and fax) of someone who will be able to check the proofs on your behalf, and who will be available to address any last-minute problems . Once your paper has been scheduled for online publication, the Nature press office will be in touch to confirm the details.

Acceptance of your manuscript is conditional on all authors' agreement with our publication policies (see www.nature.com/authors/policies/index.html). In particular your manuscript must not be published elsewhere and there must be no announcement of the work to any media outlet until the publication date (the day on which it is uploaded onto our web site).

Please note that <i>Nature Ecology & Evolution</i> is a Transformative Journal (TJ). Authors may publish their research with us through the traditional subscription access route or make their paper immediately open access through payment of an article-processing charge (APC). Authors will not be

natureresearch

required to make a final decision about access to their article until it has been accepted. Find out more about Transformative Journals

<B>Authors may need to take specific actions to achieve compliance with funder and institutional open access mandates. For submissions from January 2021, if your research is supported by a funder that requires immediate open access (e.g. according to Plan S principles) then you should select the gold OA route, and we will direct you to the compliant route where possible. For authors selecting the subscription publication route our standard licensing terms will need to be accepted, including our self-archiving policies. Those standard licensing terms will supersede any other terms that the author or any third party may assert apply to any version of the manuscript.

In approximately 10 business days you will receive an email with a link to choose the appropriate publishing options for your paper and our Author Services team will be in touch regarding any additional information that may be required.

You will not receive your proofs until the publishing agreement has been received through our system.

If you have any questions about our publishing options, costs, Open Access requirements, or our legal forms, please contact ASJournals@springernature.com

An online order form for reprints of your paper is available at https://www.nature.com/reprints/author-reprints.html. All co-authors, authors' institutions and authors' funding agencies can order reprints using the form appropriate to their geographical region.

We welcome the submission of potential cover material (including a short caption of around 40 words) related to your manuscript; suggestions should be sent to Nature Ecology & Evolution as electronic files (the image should be 300 dpi at 210 x 297 mm in either TIFF or JPEG format). Please note that such pictures should be selected more for their aesthetic appeal than for their scientific content, and that colour images work better than black and white or grayscale images. Please do not try to design a cover with the Nature Ecology & Evolution logo etc., and please do not submit composites of images related to your work. I am sure you will understand that we cannot make any promise as to whether any of your suggestions might be selected for the cover of the journal.

You can now use a single sign-on for all your accounts, view the status of all your manuscript submissions and reviews, access usage statistics for your published articles and download a record of your refereeing activity for the Nature journals.

To assist our authors in disseminating their research to the broader community, our SharedIt initiative provides you with a unique shareable link that will allow anyone (with or without a subscription) to read the published article. Recipients of the link with a subscription will also be able to download and print the PDF.

nature research

You can generate the link yourself when you receive your article DOI by entering it here: <a href="http://authors.springernature.com/share">http://authors.springernature.com/share<a>.

Yours sincerely,

{REDACTED}
